# The effect of a transient immune activation on subjective health perception in two placebo controlled randomised experiments

**DOI:** 10.1371/journal.pone.0212313

**Published:** 2019-03-06

**Authors:** Anna Andreasson, Bianka Karshikoff, Lisa Lidberg, Torbjörn Åkerstedt, Martin Ingvar, Caroline Olgart Höglund, John Axelsson, Mats Lekander

**Affiliations:** 1 Stress Research Institute, Stockholm University, Stockholm, Sweden; 2 Osher Center for Integrative Medicine, Karolinska Institutet, Stockholm, Sweden; 3 Division for Family Medicine, Department of Neurobiology, Care Sciences and Society, Karolinska Institutet, Huddinge, Sweden; 4 Department of Clinical Neuroscience, Karolinska Institutet, Stockholm, Sweden; 5 Department of Physiology and Pharmacology, Karolinska Institutet, Stockholm, Sweden; 6 Department of Medicine Solna and CMM, Karolinska Institutet and Karolinska University Hospital Solna, Stockholm, Sweden; Vanderbilt University, UNITED STATES

## Abstract

**Background:**

Patient-reported outcomes predict mortality and play increasingly important roles in care, but factors that modify central measures such as health ratings have been little investigated. Building on designated immune-to-brain pathways, we aimed to determine how a short-term induced inflammation response impacts self-reported health status.

**Methods:**

Lipopolysaccharide injections were used to provoke acute systemic inflammatory responses in healthy men and women and were compared to placebo in two double-blind randomized experiments. In Experiment 1, 8 individuals (mean 24 years; SD = 3.7) received lipopolysaccharide 0.8 ng/kg once and placebo once in a cross-over design, and in Experiment 2, 52 individuals received either lipopolysaccharide 0.6 ng/kg or placebo once (28.6 years; SD = 7.1). Main outcomes were perceived health (general and current), sickness behaviour (like fatigue, pain and negative affect), and plasma interleukin-6, interleukin-8 and tumour necrosis factor-α, before and after injection.

**Results:**

Compared to placebo, lipopolysaccharide lead to a deterioration in both self-rated general (Experiment 1, b = 1.88 for 0.8 ng/kg) and current health (Experiment 1 b = -3.00; and Experiment 2 b = -1.79) 1.5h after injection (p’s<0.01), effects that remained after 4.5 to 5 hours (p’s<0.05). The effect on current health in Experiment 2 was mediated by increased inflammation and sickness behaviour in response to lipopolysaccharide injection (β = -0.28, p = 0.01).

**Conclusion:**

Health is drastically re-evaluated during inflammatory activation. The findings are consistent with notions that inflammation forms part of health-relevant interoceptive computations of bodily state, and hint at one mechanism as to why subjective health predicts longevity.

## Introduction

Subjective health measures are direct reports of perceived health status without interpretation of the response by a clinician or anyone else. The relevance of such measures has increased following a recent investigation of about 500 000 UK Biobank participants [1], showing that probing the domain of subjective health was the best predictor of mortality. Self-rated health was the strongest predictor of five-year mortality in men, and the fourth strongest in women [1], corroborating earlier findings [2, 3]. Thus, crucial aspects of health can be subjectively perceived and quantified beyond what is obtained in core biomedical practice. The underpinnings of self-rated health have received increasing attention but in spite of an increasing number of observational studies [4], assumed biological mechanisms of perceived health have not been investigated experimentally.

Self-rated health is central among measures currently introduced to support value based health care, in which patient-reported outcome measures (PROMs) play a key role [5–7]. PROMs of subjective health need further characterization, especially since they may be integrated into future payment models [8]. Robustness, context-dependence and external validity need to be determined experimentally.

Epidemiological evidence that subjective health measures robustly predict objective health outcomes contrasts with the fact that main correlates to self-rated health, such as fatigue, pain and mood [9], fluctuate over time. While knowledge exists on behavioural determinants of self-reported health measures, biological mechanisms that influence perceived health and connect it to mortality are less well known. Cross-sectional and observational studies have however demonstrated associations between higher levels of inflammatory mediators and poor self-rated health [10–13], supporting the notion that health appraisal is influenced by body-to-brain inflammation-related cues. However, reversed causality, personality or genetic factors could also explain these associations.

Fatigue, pain and low mood are not only related to self-rated health [9], but also to central components of sickness behaviour, a term used for the coordinated behavioural response that follows immune activation [14–17]. Sickness behaviour is triggered by pro-inflammatory cytokines in response to infection or injury [17] and includes fatigue, increased pain sensitivity, worsened mood and anhedonia, anorexia, reduced social interaction, and cognitive and motivational changes [16]. When unabated, the ensuing immune signalling may contribute to chronic disorders such as depression [17]. Functional changes in brain areas involved in emotions [18], pain processing [19] and interoception (internal body perception) [15], have been demonstrated during immune activation, and constitute proposed neural mechanisms for the malaise connected with sickness behaviour.

Sickness behaviour can be studied experimentally by injecting lipopolysaccharide (LPS), a bacterial endotoxin. LPS mounts a transient systemic inflammatory response with increased concentrations of cytokines such as tumour necrosis factor (TNF)-α, interleukin (IL)-6 and IL-8 [14]. LPS-induced inflammation causes sickness and affects symptoms correlated with self-rated health, and thereby may modify central PROM constructs as perceived health. The aim of the present study was to determine how a transient inflammatory activation affects health ratings. This was investigated in two independent placebo controlled experiments. We hypothesized that health would be rated as poorer during the active phase of inflammatory activation after injection of LPS as compared to placebo. We furthermore hypothesized current health to be more strongly affected by LPS compared to general (global) health. Lastly, the hypothesis that LPS-stimulated inflammation affects perceived health through the degree of sickness behaviour it induces was tested in path models.

## Materials and methods

### Participants

For details of methods and materials, see [14]. Healthy participants were recruited by advertising and underwent a health examination with questionnaires. The participants were 18–50 years, had a normal body mass index and had to be right-handed, medication free, non-smoking, free of chronic disease and without any history of drug abuse, inflammatory, psychiatric or sleep disorders, or chronic pain. The majority of the participants were university students and were from Northern Europe. Participants were asked to sleep regular hours and refrain from alcohol and heavy exercise the day before the experiment.

### Study protocol

**Fig 1:**
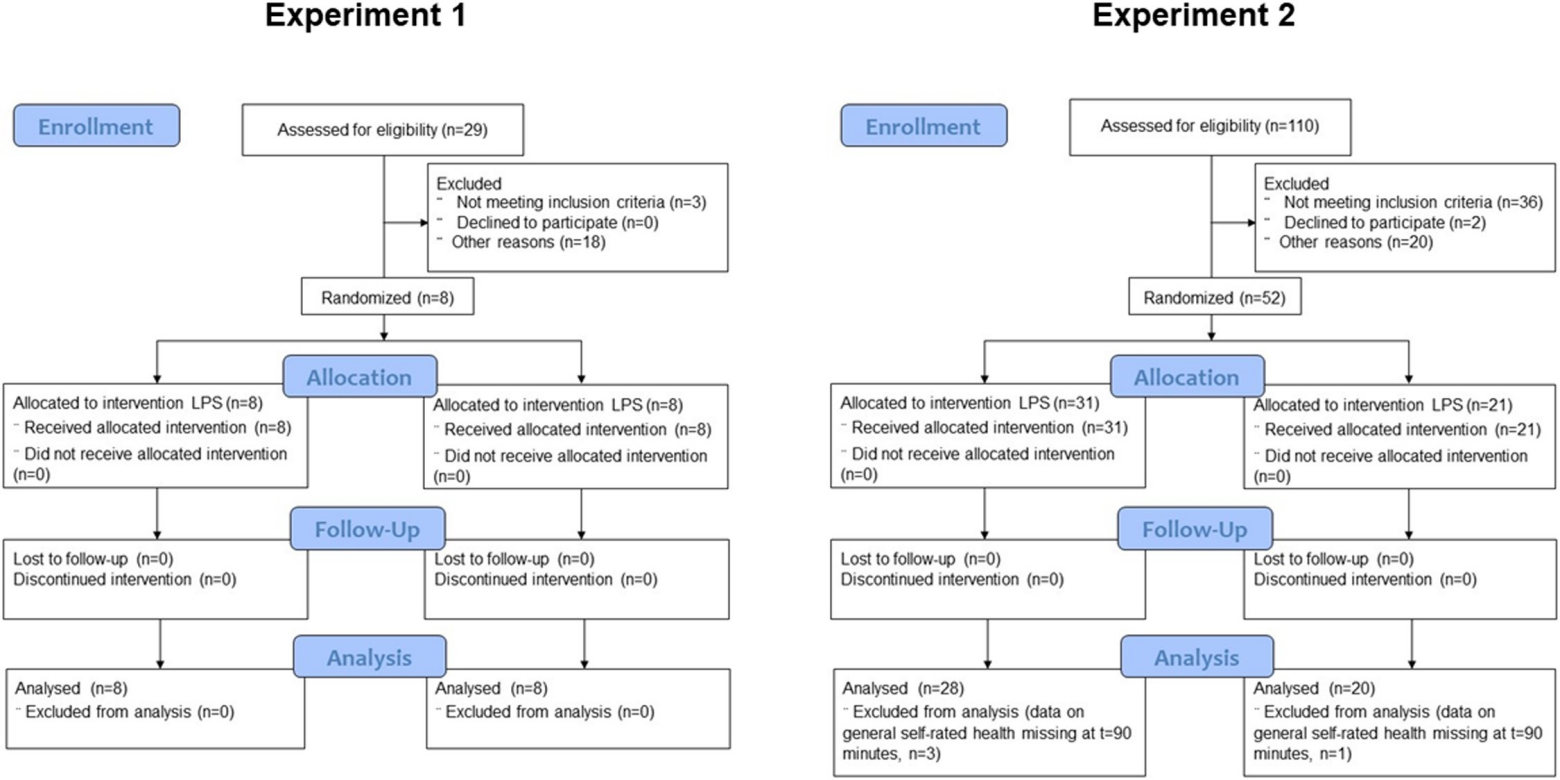
CONSORT flowcharts for experiment 1 and 2.

Both experiments were double-blind and randomized, but differed in dose, design and timing of tests [14], and were performed in a university hospital setting (Karolinska University Hospital MR center). In Experiment 1, 8 healthy participants (7 men) were injected intravenously (i.v.) twice 28 days apart, once with active substance 0.8 ng/kg body weight LPS (*Escherichia coli*, Lot nr G3E0609, United States Pharmacopeia Rockville, MD) and once with saline (placebo) in a within-subject cross-over design. The participants were randomly allocated 1:1 by simple randomization using sealed envelopes and the syringes were prepared by a nurse that did not interact with either participants or investigators. Participants were continuously monitored by a nurse and a physician. Experiment 1 was performed October to December 2009. In Experiment 2, 52 healthy participants were injected i.v. with either 0.6 ng/kg LPS (n = 31, 18 women) or saline (n = 21, 11 women) in a between-subject (Experiment 2) (overview in Table 1). The participants were randomised as in Experiment 1 using a 1:1.5 placebo:endotoxin allocation. Experiment 2 was performed October 2010 to March 2011. The participants were informed that the participation was voluntary and that they had the right to discontinue participation at any time and would then be given a non-steroid-anti-inflammatory drug to alleviate any symptoms. The responsible physician was not blinded for safety reasons; all other study personnel were blinded. None of the participants withdrew from the study. The regional ethics board in Stockholm approved the experiments and all participants gave written informed consent. The experiments were registered in ClincialTrials.gov (NCT03551080 and NCT03551184) ex post facto as they were not testing health outcomes of a treatment in symptomatic individuals but the effect of a known physiological response in healthy individuals on outcomes such as pain sensitivity, related changes in brain activity and subjective health perception. CONSORT flowcharts are presented in Fig 1.

**Table 1:** Overview of experiments and timings of sampling.

	Experiment 1	Experiment 2
Design	RC Cross over	RC Parallel
LPS dose	0.8 ng/kg	0.6 ng/kg
N	8 saline [placebo] 8 LPS	21 saline [placebo] 31 LPS
Blood samples	+ 90 min, + 240 min	+ 90 min, + 270 min
Current self-rated health	+ 90 min, + 300 min	+ 90 min, + 270 min
General self-rated health	+ 90 min, + 300 min	+ 90 min
Women (no.)	1	29
Men (no.)	7	23
Age (mean, SD)	24 ± 3.7 years	28.6 ± 7.1 years

min = number of minutes after the LPS/placebo injection, LPS = lipopolysaccharide, RC = randomized controlled

Blood was drawn via an i.v. catheter at baseline (10 am after 30 min rest), at 1.5 h and at 4 or 4.5 h (for each experiment, respectively) after injection. TNF-α, IL-6 and IL-8 plasma levels (S1 Table) were analysed as previously described [14].

#### Health ratings

Two ratings of health, general and current, were used to differentiate effects of inflammation on ratings of”trait” vs”state” health. General health was assessed using the question “How would you rate your general state of health?”, rated on a five-point Likert scale (1 = very poor, 5 = very good). General health was measured at baseline, at 1.5 h and 4.5 h after injection in Experiment 1, but only at 1.5h after injection in Experiment 2. This was done to obtain a rating of general health unbiased from other ratings the same day. Current health was assessed in both studies before injection, at peak inflammation (1.5 h post-injection) and at 4.5/5 h after injection in the respective experiment, see Table 1. The question was phrased “How is your health right now?”, rated on a seven-point Likert scale (1 = very poor, 7 = excellent).

#### Sickness behaviour

In Experiment 2, sickness behaviour was assessed with the Sickness Questionnaire [20] at baseline and 1.5 h and 4.5 h after injection. The questionnaire measures general features of sickness behaviour and consists of 10 items each scored on a four-point Likert scale (0–3), with higher points corresponding to greater sickness.

### Statistical analysis

No power calculation was made prior to Experiment 1 (with an LPS dose of 0.8 ng/kg body weight) with health as an outcome. The resulting effect sizes of the within-subject changes, was 1.24 for general health (based on z-statistics due to the ordinal properties of the scale), and 1.96 for current health. Based on similar standard deviations (1.5 hours post injection), a power of 0.80 and an alpha level of 0.05, it was estimated that we in Experiment 2 needed 27 participants for testing general health in a between-subject design (and a 60/40 rate of LPS/placebo), and 19 participants for testing current health. Experiment 2 encompassed 52 individuals, compensating for a slightly lower dose of LPS (0.6 rather than 0.8 ng/kg body weight). The choice of having an injection rate of 60%/40% for LPS/placebo was to compensate for an expected larger variation in the experiment group and to allow for analyses of individual differences in response to LPS. Experiment 2 used a lower dose of endotoxin than the pilot study and almost twice the sample size for general health, 31 participants receiving endotoxin and 21 participants receiving placebo, was included.

First, the effect of experimental immune activation on health ratings over time was tested. Repeated measures of general health (Experiment 1) and current health (both experiments) were analysed using a mixed effect regression analysis. Self-rated health data was missing for one data point in Experiment 1 (at time point 5 h in LPS condition) and for five data points in Experiment 2 (time point 1.5 h: three observations for general health, one in placebo group and two in LPS group, and two observations for current health in LPS group; time point 4.5 hours: one in the placebo group and one in the LPS group). As only eight participants participating twice are included in Experiment 1, the results from the mixed effect regression analyses were confirmed using the non-parametric and conservative Wilcoxon signrank test for the change in health perception between baseline and 90 minutes between the LPS condition and the placebo condition. In Experiment 2, the difference in self-rated general health between the LPS-injected group (n = 28) and the placebo group (n = 20) was tested using Mann-Whitney rank sum test as general health was only assessed once in Experiment 2. To test for possible gender effects, the mixed-effects linear regression model with current health in Experiment 2 was rerun with the addition of an interaction term between condition and gender). Second, the effects of experimental immune activation on current versus general health in Experiment 2 were compared, using logistic regression with general and current health as independent variables and condition (LPS vs placebo) as dependent variable. The Spearman rank sum test was used to investigate the association between ratings of general and current health in the LPS group in Experiment 2 at 1.5 h after injection. Stata 15 (StataCorp LLC, College Station, Texas, USA) was used for these analyses.

Last, the pathways from experimental immune activation to current health (for which change variables were available) via inflammation and/or sickness behaviour were tested using path analyses implemented in Mplus 7.11. The model tested the hypothesis that experimental immune activation increases inflammatory cytokines, which in turn increases sickness behaviour, ultimately causing a deterioration of self-rated health. Cytokine levels were missing from one participant throughout the experiment in the LPS group and sickness behaviour was missing from four participants at baseline (two in the LPS group and two in the placebo group) and three participants (two in the LPS group and one in the placebo group) at 1.5 h. Data from 46 participants with complete data on all included variables from Experiment 2 at baseline and 1.5 hours were included. Ranked values of the relative change in TNF-α, IL-6 and IL-8 were included as a latent variable representing inflammation and delta values for change in sickness behaviour and current health between baseline and 1.5 h were used. Standardized path coefficients (β) are reported along with two-tailed p-values. Both conventional p-values and p-values bootstrapped with 2000 repetitions were calculated. The fit of the model to the observed variance-covariance structure was assessed using several metrics including the residual Chi-Square test (ideally p>0.05), the ratio of Chi-Square to degrees of freedom (ideally <5.0), the comparative fit index (CFI, ideally >0.95) and the Tucker-Lewis index (TLI, ideally >0.95) [21].

An α-level of 0.05 was set for all statistical tests.

## Results

### Changes in perceived health over time during experimental immune activation

**Fig 2:**
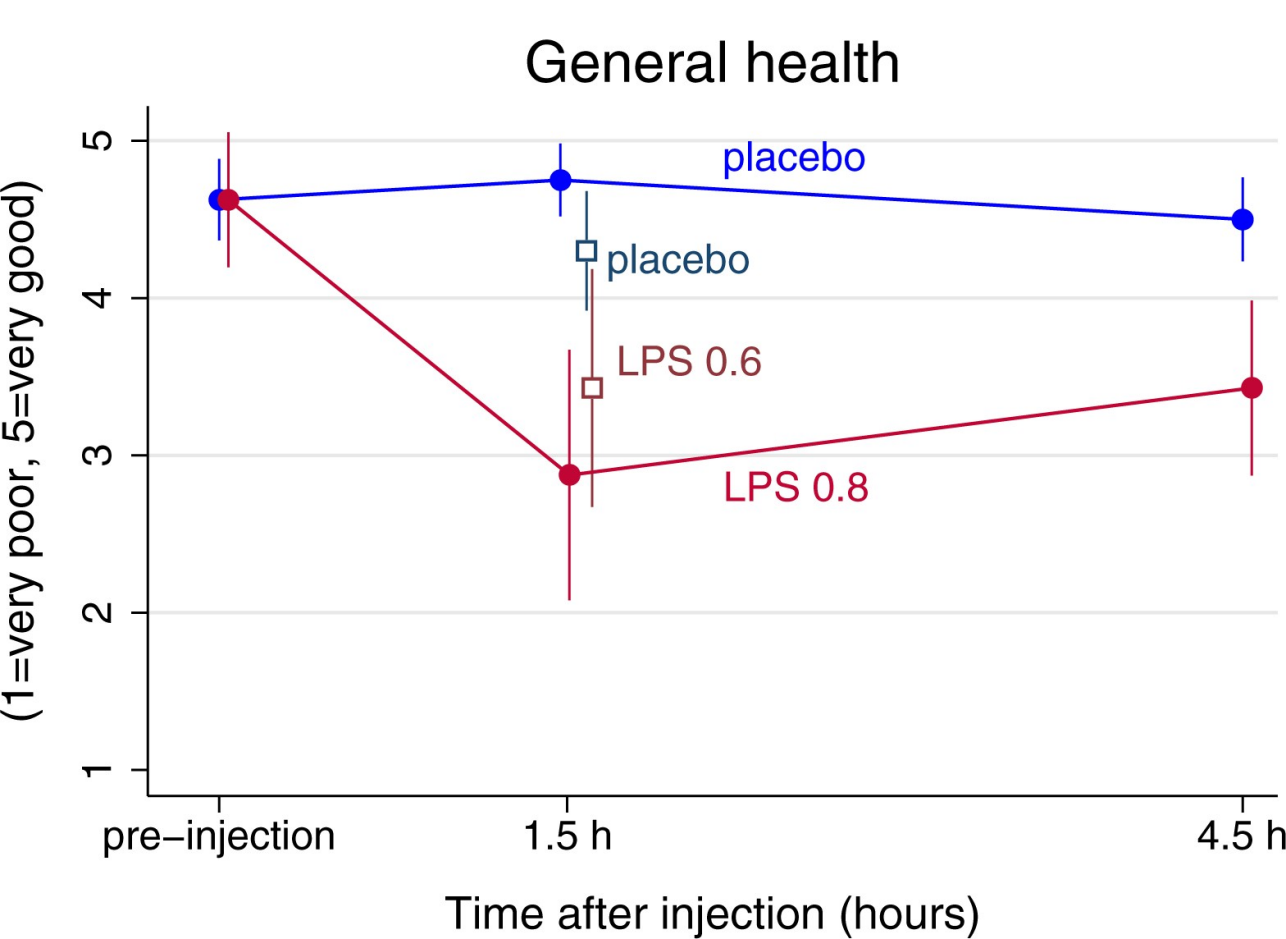
Changes in general self-rated health in response to lipopolysaccharide (LPS) and placebo. In Experiment 1, measures were made prior to, 1.5h, and 4.5h after an LPS injection of 0.8ng/kg body weight (a solid red line with filled circles) or placebo (saline, a blue solid line with filed circles). In Experiment 2, measures were made 1.5h post injection with an LPS injection of 0.6ng/kg body weight (non-filled red/marron squares) and placebo (non-filled navy blue squares) only. Means and standard error are depicted. Note that the y-scale is reversed, 1 = very good, 5 = very poor.

General health was significantly poorer in the LPS than in the placebo condition both 1.5 h (p = 0.007) and 4.5 h after injection in Experiment 1 (p = 0.01), shown by significant interactions between condition and time (Tables 2 and S1 and Fig 2). This effect was also found in Experiment 2, where general health was significantly poorer in the LPS vs. the placebo group 1.5 h after injection (p = 0.004, Mann-Whitney U-test; Fig 2). The results from Experiment 1 were corroborated by the Wilcoxon sign rank test (p = 0.03 for general health and p = 0.02 for current health).

**Table 2:** Estimates of effects on general and current health by experimental immune activation with LPS versus placebo, and time.

	Intercept	Condition (LPS) b (95% CI)	Time1 (1,5h vs. pre injection) b (95% CI)	Time2 (4.5/5h vs. pre injection) b (95% CI)	Condition×Time1 (1.5h) b (95% CI)	Condition×Time2 (4.5h/5h) b (95% CI)
General health (Experiment 1)	4.63 (4.15;5.10)	0.00 (-0.62;0.62)	0.13 (-0.50;0.75)	-0.13 (-0.75;0.50)	-1.88 (-2.76;-0.99)***	-1.10 (-2.00;-0.20)*
Current health (Experiment 1)	6.64 (6.16;7.09)	-0.25 (-1.05;0.55)	0.00 (-0.65;0.65)	-0.38 (-0.99;0.24)	-3.00 (-4.44; -1.56)***	-1.59 (-2.87;-0.31)*
Current health (Experiment 2)	6.24 (5.77;6.70)	0.02 (-0.39;0.43)	-0.62 (-1.09; -0.15)*	-0.22 (-0.70;0.26)	-1.79 (-2.47; -1.10)***	-0.91 (-1.56;-0.25)***

Estimates presented as mixed regression model (b) coefficients (95%CI). The intercept corresponds to mean health in the placebo group at baseline. The fixed effect (condition) represents any differences in health from that of the placebo group at baseline. The interaction effects accounts for differences between conditions at peak inflammation (1.5h after injection and at the end of experiment; 4.5 h after injection for Experiment 1 and at 5 h for Experiment 2). The fixed effects presented are adjusted for all other effects included in the model.

*p < .05,

***p < .001;

p-values based on 2000 bootstrap repetitions. LPS = lipopolysaccharide.

**Fig 3:**
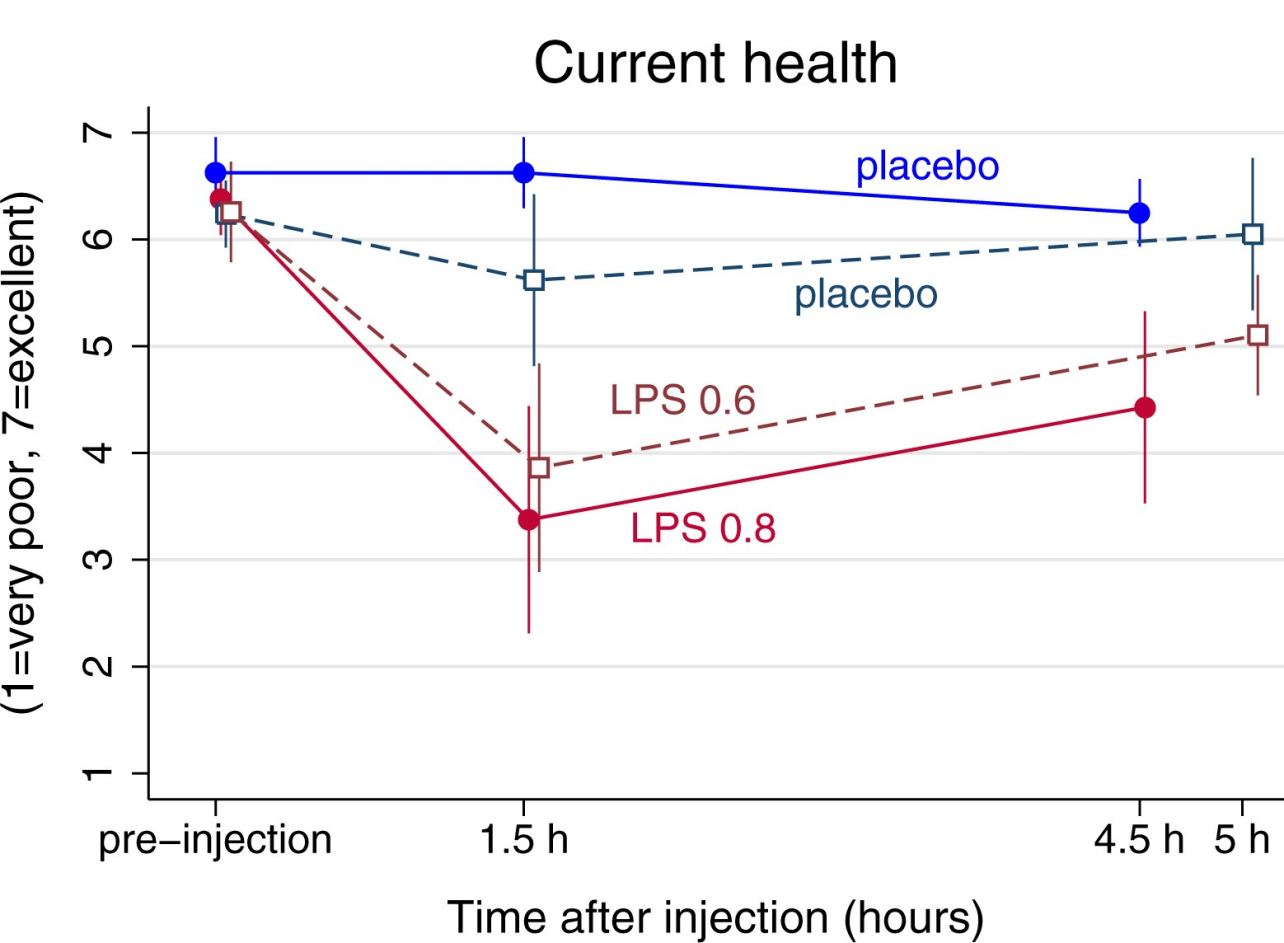
Changes in current self-rated health in response to lipopolysaccharide (LPS) and placebo. In Experiment 1, measures were made on several time points prior and post to a LPS injection of 0.8ng/kg body weight (a solid red line with filled circles) or placebo (saline, a blue solid line with filled circles). In Experiment 2 injection with an LPS injection of 0.6ng/kg body weight (a red/marron dashed line and non-filled squares) and placebo (a navy blue dashed line with un-filled squares). Means and standard errors are depicted. 1 = very poor health, 7 = excellent health.

Correspondingly, current health was significantly poorer after LPS vs placebo, both 1.5 hours after injection (p’s≤0.001 in both experiments), and 4.5/5h post injection (p = 0.005, Experiment 1; p<0.001, Experiment 2) (Fig 3 and Tables 2 and S1). A significant main effect of time in Experiment 2 showed that current health was poorer at 1.5 h after injection with either LPS or placebo (p = 0.01), but this was not found in Experiment 1. The test of the interaction between condition and gender showed no gender difference for the effects of LPS injection on health ratings (not shown) why gender was not included as an effect modifier in any of the analyses.

### Relation between current and general health

The logistic regression including condition and both current and general health showed that the LPS injection affected current health independently of general health (OR = 0.51, 95%CI = 0.29;0.90, p = 0.019), while there was no significant effect of LPS on general health independent of current health (OR = 0.62, 95%CI = 0.24;1.61, p = 0.33). General and current health ratings were significantly associated in the LPS group (n = 29, ρ = 0.48, p = 0.01) at 1.5h after injection in Experiment 2.

### Path model

**Fig 4:**
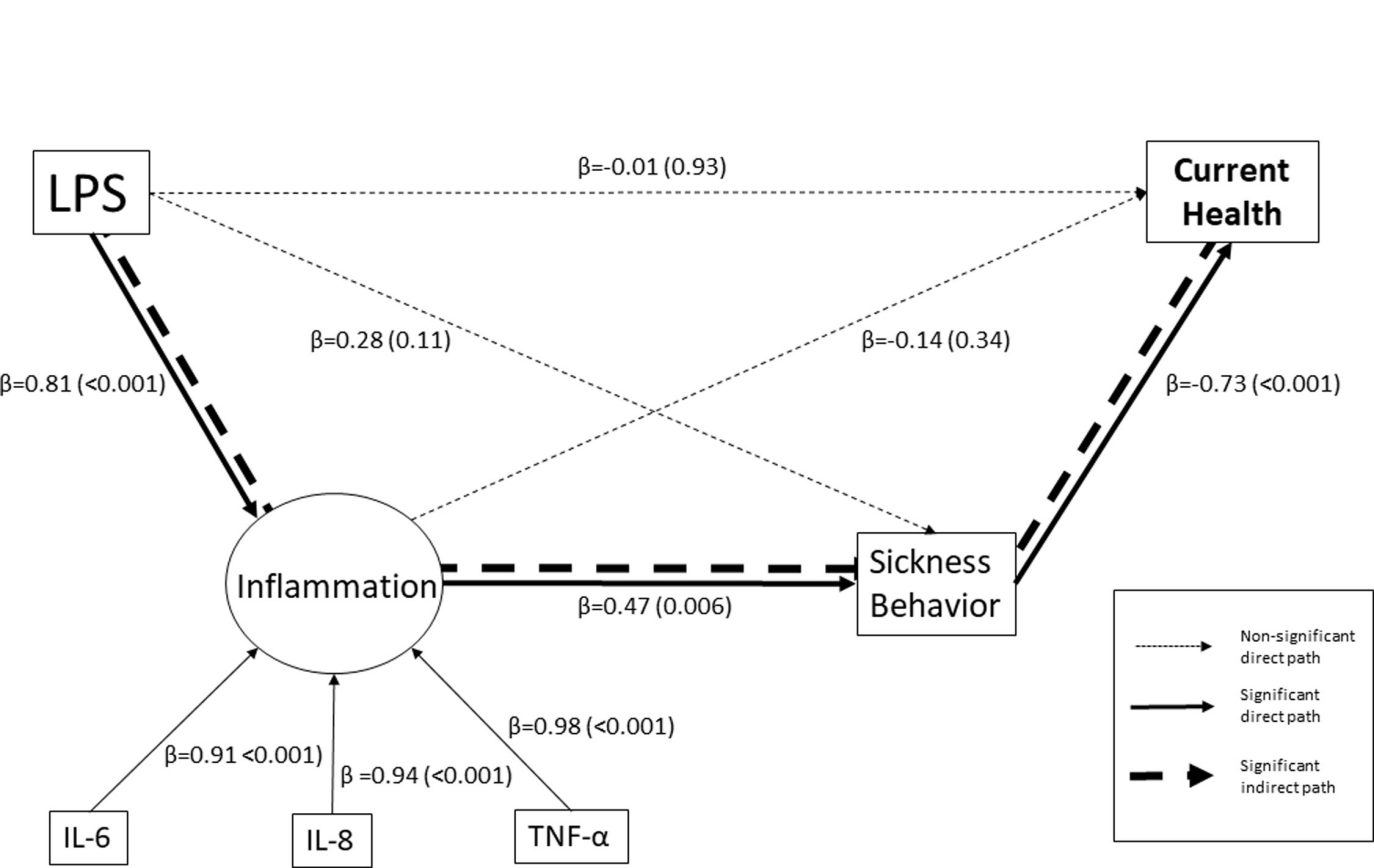
Path analysis for current health. The model describes the relationship between experimental immune activation with LPS, increase in inflammation, increase in perceived sickness behaviour and a poorer self-rated current health status, between baseline and peak inflammatory activity 1.5h after injection with LPS/placebo (n = 46). Direct paths are adjusted for the other variables presented in the model and are depicted with continuous lines, significant paths are depicted with bold lines. There was a significant indirect path connecting LPS with change in current SRH via change in inflammation and change in sickness behaviour, depicted with a bold dashed line.

The path model (Fig 4) had adequate fit with the residual Chi-Square test p = 0.18, the ratio of Chi-Square to degrees of freedom = 1.50, CFI = 0.99, and TLI = 0.98. Conventional and boot-strapped p-values were similar (conventional p-values are reported). The path analysis showed that the LPS injection resulted in poorer current health (total effect β = -0.61, p<0.001) via its indirect effect on inflammation to sickness behaviour to current health (β = -0.28, p = 0.01, Fig 4). The other paths, going directly via inflammation (β = -0.11, p = 0.35) or via sickness behaviour (β = -0.20, p = 0.11) respectively, were not significant, and neither was the direct effect of LPS injection on current health (β = -0.01, p = 0.93). Hence, it was supported that stronger LPS-induced inflammation lead to poor current health via sickness behaviour.

## Discussion

We show that experimentally induced immune activation strongly affects health ratings. The findings were robust over two experiments with different designs, and are in line with previous studies showing cross-sectional relationships between higher inflammatory activity and poor subjective health [10–13]. In agreement with the hypothesis, current health was more sensitive to change compared to general health. In addition, our results indicate that health ratings were primarily based on sickness behaviour, supporting that the mechanisms connecting systemic inflammation to relatively poorer ratings of health involve classical sickness symptoms such as fatigue, depressed mood, and pain. In sum, the findings from the present study provide experimental support for the notion that inflammation can affect self-rated health, and suggest that the degree of sickness induced by immune-to-brain-signalling is involved in the chain of mechanisms.

Given that subjective health ratings predict future objective health [1, 3], and that the general use of PROMs increases cost-effectiveness and improve survival in health care [5, 7], a mechanistic perspective is warranted. It is thus important to understand how PROMs are related to inflammatory activation both in situational (affecting ratings) and long-term (connecting to future health) perspectives. Low-level inflammation is in fact involved in many of today’s disorders, like metabolic problems, vascular disorders, depression and solid tumour cancer [22, 23], but seems possible to influence by changed life style [24, 25].

Earlier cross-sectional studies have demonstrated correlations between higher levels of inflammatory markers and poorer self-rated health [10–13], and the present results show that these variables can be causally connected in a short-term time frame. None the less, a recent study showed that stable general good health (between-person differences), but not variation in health (within-person changes), correlated consistently with low IL-6 over time in older adults [11]. In that study, the time scale of measurement discrepancies in health ratings and IL-6 was days to weeks. We therefore suggest that the association between inflammation and perceived health has a trait-like component in addition to a state-like relationship over very short time scales (hours to days). Notably, the underpinnings of self-rated health is of a multifactorial nature (see [4]) and the present experimental design cannot answer questions regarding reversed causality in long-term disease processes and health development. For example, self-rated health may be coupled to future health through influences on behaviour [2] and possibly on inflammatory markers through known pathways of neuroendocrine regulation of inflammatory processes [17, 26].

While changes in current health are intuitive in a model of acute inflammation, the present study convincingly demonstrates effects of inflammation on how a person reports her general state of health. In spite of being young and healthy, health was during inflammatory activation rated at a level similarly to previously studied middle-aged primary care patients [12]. In consonance with our hypotheses, this shows that health appraisals are generally sensitive to short-term changes in internal bodily state. The stronger effect in Experiment 1 compared to Experiment 2 might reflect a dose-response relationship between degree of change in bodily state and effect on health ratings.

The exact wording when measuring perceived health has previously been shown to be of minor importance for its predictive validity [2, 27]. However, the importance of the time frame for which health is considered has been less investigated. It is likely that current health more closely follows the development and recovery of immune activation than general health, an assumption supported by our data. In turn, it is likely that an individual’s evaluation of his/her general health condition is influenced by the current state, a notion which is likewise supported by our data.

Sickness behaviour is primarily induced by pro-inflammatory cytokines. During the response, the inflammatory activity in the brain increases and modulates neural activity [28, 29], which is believed to induce the sensations of malaise [17, 19]. In this process, interoceptive pathways including the insula are activated [15, 19]. Because these pathways are central in producing awareness of different bodily states [30], it is hypothesized that health appraisal is influenced by sickness signals within this interoceptive system, specifically so in terms of the degree of behavioural changes the signal results in. In addition to general feelings of sickness, it is also possible that poorer health ratings during inflammatory activation are due to a negative bias connected to dysphoria and changed motivational priorities displayed during sickness [17].

The main strengths and weaknesses of this study stem from the experimental model where the effects of a relatively mild and only transient systemic immune response were studied in healthy young participants. The controlled setting entails obvious advantages. We could largely isolate and describe one specific aspect of perceived health, an immune system-driven afferent component, and this was done in two different experiments with different designs and doses, and both in men and women. This notwithstanding, there is a need for further experiments to understand the causal pathways between immune activation and health perception. Such knowledge may shed light both on potential biases in application of PROMs in health care systems, but can also inform interventions to improve perceived health.

A few data points for self-rated health was missing from LPS injected participants which may have induced bias. However, the missing ratings were likely from those participants more strongly affected by LPS which could indicate that the effect of a transient immune activation might be stronger than demonstrated in the present studies.

In conclusion, we show in two experiments that a transient systemic inflammatory activation affects how people judge their health status. We found support for a model where inflammation-driven sickness is an important mediator for the impact on health ratings. We argue that experimental and mechanistic approaches will contribute not only to an increased understanding of perceived health and the importance of PROMs, but ultimately also give insights into the strong relation between subjective health and future morbidity and mortality [1, 2, 4].

## Supporting information

S1 Table Mean levels and 95%CI of general health, current health, sickness behaviour, and cytokines in experiment 2 (1.5h after Injection). (DOCX)

S1 File CONSORT checklist. (DOC)

S2 File Original ethics application with approval in Swedish. (PDF)

S3 File Original ethics amendment 1 in Swedish. (PDF)

S4 File Original ethics amendment 2 in Swedish. (PDF)

S5 File English template ethics review board application form. (PDF)

S6 File English translation of ethics application. (DOCX)

S7 File English translation of ethics amendment 1. (DOCX)

S8 File English translation of ethics amendment 2. (DOCX)
