# Supplementary material for: The effect of a transient immune activation on subjective health perception in two placebo controlled randomised experiments
Source: PLoS One. 2019 Mar 6;14(3):e0212313. doi: 10.1371/journal.pone.0212313 (PMC6402640; doi:10.1371/journal.pone.0212313)
Supplement: S1 Table — (DOCX) [file pone.0212313.s001.docx]

**Supplementary Appendix**

Table of content: Table S1 to “The effect of a transient immune activation on subjective health perception in twoa placebo controlled randomised experiments”

Investigators:

Anna Andreasson, PhD, Bianka Karshikoff, PhD, Lisa Lidberg, MSci, Torbjörn Åkerstedt, PhD, Martin Ingvar, PhD, Caroline Olgart Höglund, PhD, John Axelsson, PhD, Mats Lekander, PhD

## **Table S1. Mean Levels and 95%CI of General Health, Current Health, Sickness Behaviour, and Cytokines in Experiment 2 (1.5h after Injection)**

|  |  | LPS |  |  | Placebo |  |
| --- | --- | --- | --- | --- | --- | --- |
|  | n | Mean | 95%CI | n | Mean | 95%CI |
| General health | 28 | 4.30 | 3.99-4.61 | 20 | 3.43 | 3.01-3.84 |
| Current health | 29 | 3.86 | 3.34-4.39 | 21 | 5.62 | 4.98-6.25 |
| Sickness behavior | 29 | 16.3 | 13.6-19.0 | 20 | 7.10 | 4.1-9.9 |
| IL-6 (pg/ml) | 30 | 84.5 | 44.5-124 | 21 | 4.77 | .15-9.39 |
| IL-8 (pg/ml) | 30 | 24.8 | 8.56-41.0 | 21 | 1.55 | 1.07-2.02 |
| TNF-α (pg/ml) | 30 | 65.3 | 43.0-87.8 | 21 | 3.82 | 2.88-4.76 |

LPS=lipopolysaccharide. For description of score ranges, see the Methods section of the text.
