# Supplementary material for: The effect of a transient immune activation on subjective health perception in two placebo controlled randomised experiments
Source: PLoS One. 2019 Mar 6;14(3):e0212313. doi: 10.1371/journal.pone.0212313 (PMC6402640; doi:10.1371/journal.pone.0212313)
Supplement: S2 File — (PDF) [file pone.0212313.s003.pdf]

Regionala etikprövnings-
nämnden i Stockholm

2008 -06- 13

Dnr: 2008/955 -31

Handl:

PlusGirot 20080612

GIRERING TILL KONTO 95 06 49-4

Avsändarkonto
95 61 59-8

KAROLINSKA INSTITUTET CNS, SPEC U
Z5:00 KAROLINSKA UNIV.SJH
171 76 STOCKHOLM

2008/955.-31

MATS LEKANDER.IMMUNAKTIVERING OCH HJÄRNFUNKTION

Inflammation

AVSER REFERENS 5092120200627697 URSP.BEL SEK 5 000,00 SIDA 1(1)

SLUT PÅ DENNA REDOVISNING

SLUT PÅ DENNA REDOVISNING

003323

EPN REGIONALA ETIKPRÖVNINGSNÄMNDEN STOCKHOLM

Avdelning 1

PROTOKOLL 2008/1:7
2008-07-16

Sammanträde i Stockholm

Närvarande
Olof Forssberg, ordförande
Hans Glaumann, vetenskaplig sekreterare *(infektionssjukdomar)*
Freidoun Albertoni *(cancersjukdomar)*
Göran Elinder *(barnmedicin)*, deltar inte i ärendena 2008/531 och 2008/957
Guro Gafvelin *(molekylär immunologi)*
Agneta Herlitz *(geriatrik)*, deltar inte i ärende 2008/919
Greger Lindberg *(gastroenterologi)*
Anna-Berit Ransjö-Arvidsson *(omvårdnad)*
Anette von Rosen *(kirurgi)*
Carl-Olav Stiller *(klinisk farmakologi)*
Anna Åberg Wistedt *(psykiatri)*

Elisabeth Fleetwood *(allmänföreträdare)*
Benkt Kullgard *(allmänföreträdare)*
Signe Levin *(allmänföreträdare)*
Lars Åstrand *(allmänföreträdare)*, deltar inte i ärendena 2008/919 och 2008/926

Övriga
Pernilla Asp, *administrativ sekreterare*

---

§ 1
Ordföranden förklarar mötet öppnat.

§ 2
Ordföranden förordnar Hans Glaumann, Guro Gafvelin, Agneta Herlitz, Greger Lindberg, Anna-Berit Ransjö-Arvidsson, Carl-Olav Stiller, Anna Åberg Wistedt, Elisabeth Fleetwood, Benkt Kullgard, Signe Levin och Lars Åstrand att vid detta sammanträde tjänstgöra på avdelning 1 samt utser Hans Glaumann att vara vetenskaplig sekreterare.

§ 3
Ansökningar om etisk granskning av forskningsprojekt, se **Bilaga**.

§ 4
Ordföranden förklarar mötet avslutat.

Stockholm som ovan

Olof Forssberg
Ordförande

Hans Glaumann
Vetenskaplig sekreterare

Diarienummer *Föredragande*	Utdrag ur protokoll

2008/955-31
Agneta Herlitz

Sökande: Karolinska Institutet
Behörig företrädare: Johan Forsslöf
Projekt: Inflammation och Hjärnfunktion.
Forskare som genomför projektet: Mats Lekander

BESLUT
Nämnden godkänner forskningen på villkor att

- forskningspersonsinformationen kompletteras med uppgift om hur lång tid MR-undersökningen tar samt
- informerat samtycke till behandling av känsliga personuppgifter (se förslag till information i **underbilaga 1)** och avseende biobank (se förslag **underbilaga 2)** inhämtas från forskningspersonerna.

Hur man överklagar, se särskild information.

Att utdraget överensstämmer med originalet intygar:

Anne Manninen, administrativ sekreterare
Expedierat 2008-07-28

Samtycke till behandling av känsliga personuppgifter skall föregås av information om

- vem som är personuppgiftsansvarig (organisation/huvudman i Sverige),
- ändamålen med behandlingen av personuppgifterna,
- all övrig information som behövs för att den registrerade skall kunna ta till vara sina rättigheter i samband med behandlingen såsom uppgift om mottagarna av personuppgifterna, om vilka uppgifter som behandlas samt om rätten att ansöka om information (s.k. registerutdrag) och få rättelse av eventuellt felaktiga personuppgifter.

INFORMERAT SAMTYCKE (biobank)

Undertecknad har tagit del av den skriftliga informationen.

Jag accepterar

att delta i forskningsstudien rörande................................ och har förstått att mitt deltagande är helt frivilligt och kan avbrytas när som helst utan någon förklaring

Jag godkänner

att de vävnadsprover som jag lämnar kommer att sparas i biobank vid **(/Karolinska Universitetssjukhuset/ Karolinska Institutet/ Annat ställe)**,

att proverna används på det sätt som beskrivits i forskningspersonsinformationen men att jag när som helst kan återkalla mitt samtycke till användning av mina prover och begära att proverna omedelbart förstörs eller avidentifieras,

att proverna används i framtida forskning som inte är beskriven här och som i förekommande fall kommer att granskas och godkännas av regional etikprövningsnämnd samt att i samband med sådan forskning journalkopior eller information baserad på min journal lämnas ut (stryks om försökspersonen motsätter sig det)

Datum:____

Underskrift:____

Personnummer ☐☐☐☐☐☐☐☐☐☐

Upprättas i två original varav provgivaren behåller det ena och det andra arkiveras av ansvarig prövare.

REGIONALA ETIKPRÖVNINGS-
NÄMNDEN I STOCKHOLM

Bilaga

HUR MAN ÖVERKLAGAR

Den som vill överklaga Etikprövningsnämndens beslut skall göra detta genom att skriva till den Centrala nämnden för etikprövning av forskning. Skrivelsen med överklagandet skall dock skickas eller lämnas till Etikprövningsnämnden. Den skall ha kommit in till Etikprövningsnämnden senast inom tre veckor från den dag då klaganden fick del av beslutet.

Överklagandet skall innehålla uppgifter om

1. klagandens namn, adress och telefonnummer,
2. det beslut som överklagas (etiknämndens avdelning samt dag och diarienummer)
3. den ändring i beslutet som klaganden yrkar.

Postadress	Besöksadress	Telefon	Fax	E-Mail
Box 289	Nobels v 12 A	08-524 864 00 (vx)	08 52486699	kansli@stockholm.epn.se
171 77 STOCKHOLM	Solna			

ANSÖKAN OM ETIKPRÖVNING

se anvisning sid. 16

Uppgifter som fylls i av den regionala etikprövningsnämnden

Ankomstdatum: Dnr:

Avgift inbetald datum: Begäran om komplettering av ansökan:

Ansökan komplett:

Begärd information inkommen: Begäran om ytterligare information:

Expeditionsdatum: Beslutsdatum:

Uppgifter som fylls i av sökanden

Till Regionala etikprövningsnämnden i:
(Den regionala etikprövningsnämnd till vars upptagningsområde forskningshuvudmannen hör, se www.forskningsetikprovning.se)

Projekt

Ange en beskrivande titel på svenska för lekmän, utan sekretesskyddad information.<br>Ange också i förekommande fall projektets identitet, projektets/forskningsplanens (protokollets eller prövningsplanens) nummer, version, datum osv.

Inflammation och Hjärnfunktion

Projektnummer/identitet: Version nummer:

Ansökan avser (gäller även vid begäran om rådgivande yttrande):

forskning där endast en forskningshuvudman deltar (5000 kr) ☒

forskning där fler än en huvudman deltar (16000 kr) ☐

forskning där mer än en forskningshuvudman deltar, men där samtliga forskningspersoner eller forskningsobjekt enligt 4 § lagen (2003:460) om etikprövning av forskning som avser människor, har ett omedelbart samband endast med en av forskningshuvudmännen (5000 kr) ☐

endast behandling av personuppgifter (5000 kr) ☐

forskning som gäller klinisk läkemedelsprövning (16000 kr) ☐

ändring av tidigare godkänd ansökan (enligt 4 §) (2000 kr) ☐

Om nämnden finner att studien/forskningsprojektet inte faller inom lagens för etikprövning tillämpningsområde önskas ett rådgivande yttrande

Ja: ☒ Nej: ☐

1. Information om forskningshuvudman m.m.

1:1 Sökande forskningshuvudman

Ansökan om etikprövning av forskning skall göras av forskningshuvudmannen. *Med forskningshuvudman avses en statlig myndighet eller en fysisk eller juridisk person i vars verksamhet forskningen utförs.* Inom staten utförs forskning främst vid lärosätena, men även vid vissa andra myndigheter, som t.ex. Brottsförebyggande rådet och Socialstyrelsen. Kommuner och landsting kan vara forskningshuvudmän, liksom privaträttsliga juridiska personer.

Namn: Karolinska Institutet

Adress: Karolinska Institutet, CNS, Sektionen för psykologi
Fogdevreten 2A
17177 Stockholm

1:2 Behörig företrädare

Behörig företrädare för forskningshuvudmannen (t.ex. prefekt, enhetschef, verksamhetschef). Forskningshuvudmännen bestämmer själva, genom interna arbets- och delegationsordningar eller genom fullmakt, vem som är behörig att företräda forskningshuvudmannen. Bifoga kopia av sådan handling.

.

Namn: Johan Forsslöf Tjänstetitel: Administrativ chef

Adress: Institutionen för Klinisk Neurovetenskap (CNS)
Karolinska Universitetssjukhuset, Solna
171 76 Stockholm

1:3 Forskare som är huvudansvarig för genomförandet av projektet (kontaktperson)

Namn: Mats Lekander

Adress: Fogdevreten 2A
17177 Stockholm

E-postadress: mats.lekander@ki.se

Telefon: 08-52482445

Mobiltelefon: 0703-893341

1:4 Plats

Plats/er där projektet skall genomföras (ange inrättning/ar, institution/er, klinik/er etc.).

Karolinska Sjukhuset

1:5 Andra medverkande

Övriga deltagande forskningshuvudmän samt forskare ansvariga att lokalt genomföra projektet (kontaktpersoner) skall anges i bilaga (namn, adresser).

John Axelsson
Martin Ingvar
Caroline Olgart Höglund

1:6 Vid läkemedelsprövning

Ansökan om tillstånd har insänts till Läkemedelsverket.

Ansökan inlämnad (datum): Tillstånd erhållits ☐

1:7 Vid viss genetisk forskning

Anmälan till *Datainspektionen* om förhandskontroll av behandling av personuppgifter om genetiska anlag som framkommit efter genetisk undersökning (10 § första stycket 2 personuppgiftsförordningen (1998:1191).

Inlämnad (datum): Kommer att inlämnas efter godkänd etikprövning ☐

2. Uppgifter om projektet

2:1 Sammanfattande beskrivning av forskningsprojektet (programmet)

Beskrivningen skall kunna förstås av nämndens lekmän. Undvik därför terminologi som kräver specialkunskaper. Ange bakgrund och syfte för studien samt den (de) vetenskapliga frågeställning (ar) som man söker svar på. Ange de viktigaste undersökningsvariablerna. Ange vilka kunskapsvinster projektet kan förväntas ge och betydelsen av dessa. Ange om det är en registerstudie, uppdragsforskning etc.
För fackmän avsedd detaljerad information i protokoll eller forskningsplan <u>*skall*</u> bifogas som bilaga. En utförligare beskrivning över genomförandet *avsedd för lekmän* kan vid behov bifogas den för fackmän avsedda obligatoriska forskningsplanen.

Bakgrund

När celler inom immunsystemet känner igen främmande mikroorganismer frisätter de hormonliknande signalämnen med kapacitet att aktivera en inflammatorisk process, så kallade pro-inflammatoriska cytokiner. Utöver den perifera inflammationsreaktionen påverkas även centrala nervsystemet (CNS), där det finns en hög koncentration av cytokinreceptorer i strukturer med viktiga reglerande funktioner, till exempel i hypotalamus. Djurförsök visar att stimulering av dessa receptorer medför ett flertal påtagliga beteendeförändringar, som kallas sjukdomsbeteenden.

En generell aktivering av immunsystemet medför bl. a. trötthet, ökat sömnbehov, förändrade matpreferenser, socialt tillbakadragande, ökad smärtkänslighet och försämrad minnesfunktion, vilket tillsammans betecknas som sjukdomsresponsen. Dessa beteendeförändringar påminner om de ospecifika och svårdiagnosticerade symptom som är vanligt förekommande inom t.ex. primärvården, såsom trötthet, nedstämdhet och smärtkänslighet. Trots en hög klinisk relevans finns påfallande lite experimentell forskning utförd på människor kring påverkan av perifer inflammation på hjärnan. De humanstudier som finns har koncentrerat sig på hur det aktiverade immunsystemet påverkar minne och inlärning, sömn och aptit, och i viss utsträckning ångestsymptom. Den enda humanstudien som har studerat hur ett experimentellt aktiverat immunsystem påverkar aktivitet i hjärnan fann att aktivitet i den insulära lobens bark (insula cortex), ett område mellan hjäss- och tinninglob som bl.a. registrerar förändringar i kroppens inre miljö, och den främre delen av gördelvindlingens bark (anterior cingulate cortex, ACC) är korrelerad med sämre lungfunktion och större mängd pro-inflammatoriska cytokiner hos astmatiker som provocerats med allergen. Studien tyder på att dessa delar i hjärnan är viktiga för den subjektiva upplevelsen av sjukdom och obehag som uppstår när immunsystemet aktiveras vid exempelvis en inflammation.

Vi har i flera tvärsnittsstudier visat starka samband mellan inflammatoriska markörer (cytokiner) och subjektiv hälsoupplevelse. Forskningslitteraturen visar att den cytokininducerade sjukdomsresponsen inbegriper starka beteendeförändringar, vilka tyder på en uppmärksamhetsförskjutning från yttre (perceptiva) till inre (interoceptiva; t.ex. smärta och klåda) signaler. Studier visar även på en koppling mellan inflammation och smärta. Både interoceptiv perception och smärtperception har visat sig vara knutna till aktivitet i insula cortex och ACC. Vi vill därför undersöka hur en experimentell immunaktivering med endotoxin (som är en etablerad modell för en systemisk inflammation) påverkar den subjektiva upplevelsen av ohälsa, och om en persons uppmärksamhet förskjuts mot inre signaler när immunsystemet är aktiverat. Vi vill undersöka hur smärtkänsligheten påverkas, och vi tror att aktiviteten i vissa specifika områden i hjärnan kommer påverkas av immunsvaret, närmare bestämt insula cortex och ACC. Vi menar att denna aktivering i hjärnan är del i mekanismerna bakom sjukdomsbeteendet.

I anslutning till studien kommer en pilotstudie genomföras, där förändringar i utseende och rörelsemönster under sjukdom studeras. Vi kommer att låta läkare och lekmän

studera försökspersonernas ansikte och gång på fotografi och video för att utröna om sjukdomsresponsen kan tolkas visuellt. Se projektbeskrivningen för detaljer.

Syften och vetenskapliga frågeställningar

Syftet med studien är att studera hur det aktiverade immunsystemet påverkar hjärnaktivitet (primärt ACC och insula cortex), kognition (perception av yttre kontra inre stimuli och smärta) och subjektivt upplevd hälsa. Vi vill även utvärdera om det finns ett samband mellan hur stark immunaktiveringen är och hur stor påverkan är på hjärnaktivitet och kognition, samt om eventuella könsskillnader föreligger.

De viktigaste undersökningsvariablerna

Blodprover tas för analyser av perifer immunaktivering (pro-inflammatoriska cytokiner som TNF-alfa, IL-1β och IL-6 och antiinflammatorisk aktivitet som IL-1ra). Deltagarna kommer att skatta upplevd hälsa, humör och trötthet i samband med blodprover och fMRI-undersökning. I det första kognitiva testet, som rör perception av yttre kontra inre stimuli, bedömer försökspersonen om ljudsignaler (10 st toner) presenteras i samklang med försökspersonens hjärtrytm, samt om de presenterade tonerna innehåller en tonförändring. Det andra kognitiva testet mäter om och hur immunaktivering påverkar smärtkänslighet, med hjälp av värmestimulering av handens ovansida. Det tredje kognitiva testet mäter om sjukdomsbeteende påverkar hur man reagerar på social exklusion, som är en form av ”social smärta”. Deltagaren deltar i ett virtuellt spel med två fiktiva partners och blir vid tillfällen exkluderad från spelet. Försökspersonerna fotograferas och filmas även vid ett tillfälle under försöket, och foto och video används senare för pilotstudien.

Kunskapsvinster och betydelse av resultaten

Det är mycket vanligt med diffusa symptom (trötthet, nedstämdhet, ökad smärtkänslighet, aptitförändringar) som snarare än att vara sjukdomsspecifika tycks vara gemensamma för många ohälsotillstånd och som delvis kan vara relaterade till låggradig inflammatorisk påverkan på hjärnan. Eftersom sådana symptom är determinanter för den självskattade hälsan, som är en viktig ohälsomarkör och en riskfaktor för sjukskrivning, är det angeläget att bättre förstå hur immusystemet påverkar hjärnfunktion, symptom och beteenden. Sammantaget kommer studien att kunna

generera kunskap om mekanismer för generella sjukdomssymptom. Pilotstudien syftar till att generera kunskap kring processer som påverkar hur en persons häsla uppfattas av utomstående, primärt yrkesverksamma inom vården. Denna kunskap är högst relevant för diagnostikförfarandet på klinik.

2:2 Vilken primär vetenskaplig frågeställning ligger till grund för projektets utformning

Om projektet kan karakteriseras som en hypotesprövning, ange den primära och eventuellt sekundära hypotesen. Hänvisning till mer detaljerad information för fackmän kan ske till bifogat protokoll eller forskningsplan enligt 2:1

Våra primära hypoteser är att immunaktivering bidrar till en omställning av uppmärksamhetsfokus från yttre till inre signaler vilket resulterar i ökad bearbetning av interoceptiv (hjärtslag) jämfört med perceptuell (tonhöjd) information. Likaså tror vi att immunaktivering ger ökad smärtkänslighet samt ökad känlighet för social exklusion. Vi förväntar oss även att immunaktivering tillfälligt ger en sämre upplevd subjektiv hälsa. Mekanismen bakom dessa förväntade effekter av immunaktivering tror vi är en ökad aktivitet i relevanta områden i hjärnan, primärt insula cortex och ACC.

2:3 Redogör för resultat från relevanta djurförsök

För viss, främst medicinsk, forskning ange skälen till att djurförsök inte utförts.

Djurstudier har visat att beteende och hjärnfunktion påverkas av perifer experimentell immunstimulering, och även direkt tillförsel av cytokiner i centrala nervsystemet. När djur behandlas med endotoxin (bakteriella restprodukter som känns igen av immunsystemet), har man kunnat se tecken på depressivt beteende samt ett sjukdomsbeteende, såsom tillbakadragenhet, mindre social interaktion, lägre aptit, mindre utforskande beteende samt förhöjd smärtkänslighet. Stimulering har visats aktivera t.ex. amygdala och hypotalamus. Begräsningen i djurstudier är att beteendet kan vara svårt att tolka och överföra till människa, samt att lätt immunaktivering är svår att studera hos djur då beteendeförändringarna ofta inte är tillräckligt tydliga. Av denna anledning har betydligt högre doser av endotoxin använts än i studier på människa. I denna studie intresserar vi oss i första hand om den subjektiva upplevelsen av sjukdom, och den frågeställningen är inte möjlig att undersöka i djurexperiment.

2:4 Redogör översiktligt för undersökningsprocedur, datainsamling och datas karaktär

Av beskrivningen skall framgå hur studien planeras genomföras. Beskriv insamlade datas karaktär. Hur säkerställs datas tillförlitlighet (t.ex. kvalitetskontroll/monitorering)? - Vid enkäter och intervjuer skall beskrivas tillvägagångssätt och t.ex. frågors innehåll och hur slutsatser dras. Enkäter och skattningsskalor skall bifogas.<br>- För medicinsk forskning skall anges t.ex. typer av ingrepp, mätmetoder, antal besök, tidsåtgång vid varje försök, doser och administrationssätt för eventuella läkemedel och/eller isotoper, blodprovsmängd (även ackumulerad mängd vid multipla försök). Ange även om och på vilket sätt undersökningsprocedur m.m. skiljer sig från klinisk rutin. Ange proceduren för att ge den eventuella behandling efter studiens slut, som kan erfordras. Ange procedur för insamling av biologiskt material. Redogör för datakällor och procedurer vid behandling av personuppgifter. För mer detaljerad information kan hänvisning ske till bilagt protokoll eller forskningsplan enligt 2:1.

Varje deltagare deltar i studien vid två separata tillfällen, då samma protokoll används - en gång under endotoxinpåverkan, en gång med placebo (PBS). Presumtiva deltagare kommer att screenas (screeingformulär bilaga 5:1) 3-4 veckor innan eventuellt deltagande, då även ett första läkarbesök bokas. Vid läkarbesöket tas ett blodprov för C-reaktivt protein (CRP) och blodstatus med avseende på leukocyter (5 ml) . Förhöjda nivåer CRP tyder på en pågående infektion och inflammatorisk aktivitet i kroppen, och personen kan då inte delta i studien. Detta prov görs även på försöksdagens morgon. Blodstatus med differentialräkning av leukocyter visar hur många vita blodceller personen har, och vilken typ av leukocyter som finns i blodet procentuellt. Försökspersonen bör uppvisa normalinivåer. Tio till 14 dagar innan deltagande kommer försöksledaren med deltagaren att gå igenom protokollet och de skattningsskalor som ska användas. Deltagarna får även en 4 dagars sömn/hälsodagbok (bilaga 5:2) som de ska börja fylla i 2 dagar innan fMRI-mätningarna. Deltagarna instrueras att sova 7-8 timmar (inom tidsperioden 22.00-08.00) två nätter innan deltagande i fMRI-mätning för att undvika effekter av sömnbrist och dygnsrytm på kognition och immunparametrar (förhållningsregler bilaga 4:3).

Deltagarna går upp mellan 06:30 och 08:00 samma dag som testerna ska genomföras. Ett prov för CRP (5 ml) tas kl. 10.00 vid MR-centrum, och försöksledaren går igenom hälsodagboken med försökspersonen, Vid för höga CRP-nivåer i blodet, eller om fökylning eller annan sjukdom antecknats i hälsodagboken de senaste 2 dagarna, avbryts dagens studieinsamling och personen får återkomma vid ett annat tillfälle. Deltagarna instrueras att äta lunch mellan 12:00 - 13:30 och anlända till MR-centrum klockan 13.45. Deltagaren kommer att vara liggande under större delen av protokollet. 14:15 sätts en intravenös (i.v.) kateter i armvecket. Kl.15:30 tas det första blodprovet. 5 minuter senare injiceras deltagaren med endotoxin (0.8 ng/kg LPS från E.Coli) eller placebo (PBS). Från den inneliggande venkatetern tas sedan blodprov 16:30, 17:30, 18.30 samt efter att fMRI protokollet avslutats, vid 19.30. Vid varje blodprov (5 st) under studiens gång tas 17 ml blod, som sedan analyseras för cytokin- och hormonnivåer. Totalt tas alltså 90 ml blod under dagen (och sammanlagt 185 ml under hela studien). Plasmaprover sparas i -70oC för vidare analyser. Förberedelser för fMRI-mätning påbörjas vid 17:45 när tillgång till kameran startar, då den används för kliniskt bruk under dagtid. fMRI-protokollet påbörjas 18:00 och avslutas ca. 19.40. Försökspersonen får dricka vatten under hela studiens gång, och får vid två tillfällen ett lättare mål mat.

Laboratorieprotokoll (översikt)
10:00 Blodprov för CRP-analys (säkerställa deltagande). Möte med försöksledaren och genomgång av hälsodagbok. (20 min)
13.45 FP kommer till lab, tar av ytterkläder (tidsåtgång 30 min)
14:15 FP ligger ned och förbereds för sättande av i.v.-kateter (15 min)
14:30 Skattningar-hälsa för baseline (formulär 5:3) (5 min)
14:35 Vila, lättare mål mat (ca 55 min)
15:30 Blodprov och skattningar-hälsa för baseline (formulär 5:3) (5 min)
15:35 Injektion med endotoxin/placebo (5 min)
15:40 Vila (50 min)
16:30 Blodprov & skattningar-hälsa (formulär 5:3) (5 min)
16:35 Vila (55 min)
17:30 Blodprov & skattningar-hälsa (formulär 5:3) (5 min)
17:35 Fotografering och filmande av gång (10 min)
17:45 Förberedelser fMRI-protokoll (15 min)
18:00 fMRI – baseline (20 min)
18:20 Ton/hjärt-rytm del 1 (12 min)

18:32 Vila och blodprov (5 min)
18:37 Ton/hjärt-rytm del 2 (12 min)
18:49 Vila & skattning-test (formulär 5:4) (5 min)
18:54 Smärtperception (12 min)
19:06 Vila & skattning-hälsa (formulär 5:3) (5 min)
19:11 Social exklusion del 1 (6 min)
19:17 Vila (formulär 5:3) (5 min)
19:22 Social exklusion del 2 (6 min)
19:28 Blodprov och skattning-test (formulär 5:4) (5 min)
19:33 Emotionell skattning av ansikten (7 min)
19.40 Ut ur fMRI, något att äta, skattning-hälsa (formulär 5:3)

Endotoxinstimulering
Endotoxin består av lipopolysaccharider (LPS) från bakteriens cellvägg som aktiverar immunsystemet, men som inte kan orsaka bakteriell infektion. Vi har för denna studie valt LPS från E. coli stammen O:113 som använts i humanstudier framför allt i USA sedan 1976, och även i Sverige de senaste åren av Sollevi och Soops forskargrupp vid KS (Dnr: 416/98, 240/99, 269/99, 377/02, 436/03, 467/4, 1305/31). De i Sverige godkända endotoxin-studierna har använt högre doser (1.0 – 4.0 ng/kg), men vi kommer använda en dos på 0.8 ng/kg för att vi intresserar oss en mild immunaktivering som leder till subtila subjektiva uplevelser, och vi vill undvika starkare kroppsliga reaktioner hos försökspersonen. Sådana lägre doser har tidigare använts i kognitiva studier och sömnstudier. I de kognitiva studier som genomförts har primärt endotoxin från Salmonella bakterien använts och de maximala kognitiva effekterna har inträffat ca. 3 timmar efter injicering. Då olika endotoxinsorter, och även olika produktionpartier, kan ha något variende effektivitet önskar vi i en mindre förstudie studera när maximal kognitiv och subjektiv effekt uppnås med just vårt endotoxin, så att vi kan optimera vårt protokoll med ± 1-0,5 timmar (se vidare under avsnitt 3:1). Ämnet importeras från Prof. Suffredinis laboratorium vid NIH, som varit med att utveckla och testa just denna typ av endotoxin på människa (Suffredini et al. 1999). Ämnet framställs under GLP (Good Laboratory Practice) riktlinjer och transporteras i pulverform (se bilagan Drug Fact Sheet för detaljer om detta referens endotoxin). Efter blandning och spädning steriliseras infusionen med autoklavering inför injicering och är bakterie- och svampfri.

Experimentell endotoxinstimulering ger influensaliknande symptom, som feber, frysningar, ökad hjärtfrekvens, illamående, huvudvärk och muskelsmärtor. Vanliga

experimentella doser för humanstudier är 2-4 ng/ml. Lägre doser (0.2-0.8 ng/kg) påverkar inte kroppstemperatur och hjärtfrekvens i samma utsträckning, men en viss sjukdomskänsla och trötthet infinner sig 2-6 timmer efter injicering, och försvinner helt inom 24 timmar. Symptomen elimineras vid behov med ibuprofen, som ges om deltagare så önskar, men experimentet måste då avbrytas för att medicinen påverkar de parametrar vi vill undersöka.

Blodprovstagning och mätning av inflammationsmarkörer
En intravenös kateter sätts 60 minuter innan endotoxinstimulering, och blodprov tas varje timme under hela protokollet med start 5 minuter före endotoxinstimulering. Blodproverna analyseras direkt efter protokollets slut med hänsyn till blodstatus (leukocyträkning). Plasma fryses (-70 °C) och analyseras efter studiens slut med hänsyn till inflammationsmarkörer (pro-inflammatoriska markörer som TNF-alfa, IL-1β och IL-6, anti-inflammatorisk aktivitet (IL-1ra)) och hormoner (kortisol). Ämnenas koncentration i blodet kommer mätas med enzyme-linked immunosorbent assay (ELISA), som har ett känslighetsområde som innefattar både normalnivåer och starkt stimulerade nivåer av cytokiner/hormoner.

Skattningar (bilaga 5:1 - 5:4)
I samband med blodprov och efter de kognitiva testerna i fMRI-mätningen kommer deltagarna att skatta sin hälsa, samt symptom som trötthet, sömnighet, aptit, ångest, nedstämdhet m.m. Deltagarna kommer även att fylla i en sömn/hälsodagbok varje morgon och kväll (tidsåtgång 5 minuter) i fyra dagar. Vi kommer främst att använda oss av utvalda frågor från tidigare utvecklade och välvaliderade skalor för ångest, depression och stress (Spielberger State-Trait Anxiety Inventory=STAI och Hospital Anxiety and Depression=HAD), trötthet (Swedish Occupational Fatigue Inventory=SOFI), aptit (Spiegel et al., 2004) och sömnighet (Karolinska Sleepiness Scale=KSS). Vi kommer även att använda 2 skalor för att mäta självskattad hälsa, en för den övergripande hälsan (Self-rated health=SRH-5) och en som mäter hälsoupplevelsen den senaste timmen (en 7-gradig Lickertskala, Axelsson et al. in prep). Hälsoskalorna har olika tidsupplösning.

fMRI-protokoll
Det första kognitiva testet involverar test av antingen 1) en bedömning av om stimuli (10 st toner) presenteras i fas med ens hjärtrytm eller inte, 2) om någon av de 10 presenterade tonerna innehåller en tonförändring eller inte. De två testen presenteras

separat och i omväxlande ordning. Vi kommer att utgå från originalprotokoll som fås av Prof. Critchley vid University College i London, publicerat i Nature Neuroscience (Critchley et al., 2004). Testet mäter i vilken utsträckning personens uppmärksamnhet vänds mot inre eller yttre signaler.

Det andra kognitiva testet mäter smärtkänslighet med hjälp av en Medoc-TSA 2001 värmeanalysator. En metallprob som är 2x3 cm stor värmer olika punkter på handels ovansida under 12 sekunder. Temperaturen varierar mellan 40-49 grader och försökspersonen kommer skatta temperaturerna på en VAS skala (Visual Analogue Scale) där 0 betyder ”ingen värme alls” och 10 betyder ”största uthärdliga smärta”. En beräkning görs på vilken temperatur som upplevs som 5 på en VAS skalan för varje individ, och med hjälp av statiskisk analys kommer vi se om den 50% känslighetsgränsen ändrats vid endotoxinstimulering.

Det tredje kognitiva testet mäter upplevelsen av social exclusion, som är ett mått på vad som kallas ”social smärta”. Försökspersonen får spela ett datoriserat bollspel som visas på en skärm i fMRI-kameran, och man säger till dem att de spelar med två andra personer som sitter i andra rum. I själva verket är det ett förprogrammerat interaktivt bollspel. Under spelets gång börjar de två andra personerna utesluta försökspersonen ut spelet, och detta uppfattas som att man bli utstött. Studier visar att det obehag som upplevs är förknippat med neural aktivering i ACC, och aktiveringsmönstret i hjärnan överlappar till viss utsträckning det som uppstår vid fysisk smärta.

Vi förväntar oss förändringar ACC och insula cortex, vilka är involverade i introception och smärtupplevelser. Sammanfattningsvis har de kognitiva testen använts med framgång i tidigare fMRI-studier. Vi har tillgång till originalprogram och orginalprotokoll, samtliga väl beprövade.

Ett kort bedömningstest (7 min) av annan persons emotionella status kommer även göras, genom att personen får bedöma foton av ansikten med olika emotionella uttryck. Kontrasten mellan att bedöma vilket emotionellt uttryck en person har och om jag tycker om personen testar mekanismen för kortikal kontroll ev. emotionell aktivering i limbiska systemet (Nyberg/Öhman, in submission).

Vi kommer även att genomföra en mindre förstudie för att säkerställa procedurer och fastställa det slutliga protokollet. En testomgång (5-8 försökspersoner) kommer

genomföras där försökspersonen stimuleras med endotoxin och får svara på samtliga frågeformulär och göra testerna, men ej delta i MR-scanning, för att se den exakta tidåtgången för formulären och att mängden frågor är hanterbar för försökspersonen. Vi vill i denna del av förstudien även fastställa den optimala timingen för maximal sjudomsrepsons, med en exakthet på 1-0,5 timmar. Då inga tidigare endotoxinstudier haft sitt huvudfokus på just sjukdomsresponsen och den subjektiva upplevelsen, är det av vikt att säkerstella att denna sammanfaller med MR-scanningen på bästa möjliga sätt. En andra omgång (5-8 försökspersoner) kommer utföras med MR-scanning, men utan endotoxin stiumering, för att fastställa den exakta timingen för fMRI protokollet.

2:5 Redogör för om insamlat biologiskt material kommer att förvaras i en biobank

Med biobank avses biologiskt material från en eller flera människor som samlas och bevaras tills vidare eller för en bestämd tid och vars ursprung kan härledas till den eller de människor från vilka materialet härrör.

Redogör för var och hur prover som skall sparas förvaras, kodningsprocedurer och villkor för utlämnande av prover. Observera att i förekommande fall skall anmälan av biobank ske till Socialstyrelsen enligt lagen (2002:297) om biobanker i hälso- och sjukvården m.m.

Kodnyckel skapas och ett kodnummer ges till varje deltagare. All insamling av data kommer att registreras med kodnummer och kan inte kopplas till deltagaren utan kodnyckel. Undantaget är blodstatusprover och CRP-prov som lämnas för akutanalyser vid kemlab på KS - där används samma procedurer som vid klinisk verksamhet (personnummer för bestämmande av identitet). Plasma från alla blodprover som tagits under eftermiddagen på försöksdagen kommer att förvaras i -70°C frys på Karolinska Sjukhusets område, märkta med kodmunner, betingelse (A/B) och tidpunkt för blodprovet. Resultaten från blodstatusproverna kommer att förvaras inlåsta vid sektionen för Psykologi, Klinisk Neurovetenskap. De kommer därefter att kodas enligt kodnyckel. Kodnyckeln kommer inte att förvaras på dator utan att finnas i pappersversion som kommer att förvaras inlåst. Även en CD-kopia kommer att skapas och förvaras brandsäkert. Inga prover kommer att utlämnas till obehörig. Vid önskemål kommer varje individ att få svar på sina egna resultat.

2:6 Redovisa tillgång till nödvändiga resurser under hela projektets genomförande

Ange vilka som har ansvaret (prefekt, verksamhetschef eller motsvarande) för forskningspersonernas säkerhet vid alla enheter/kliniker där patienter ingår samt att erforderliga ekonomiska och personella resurser finns tillgängliga. Intyg från dessa skall bifogas.

Studien genomförs inom ramen för anslag och FoUUmedel. Nödvändiga resurser tillhandahålls av Institutionen för Klinisk Neurovetenskap och MR-centrum.

2:7 Journalföring, registrering och hantering av data

Redogör för hur undersökningsprocedurer och eventuella ingrepp journalförs. Ange hur registrering och behandling av resultaten skall gå till. Om materialet skall kodas, ange proceduren, vem som förvarar kodlistor och vem eller vilka som har tillgång till dem, var de förvaras, hur länge samt om materialet kommer att anonymiseras eller förstöras. Används band- och videoinspelningar? Vilken tillgänglighet har datamaterialet? Hur förvaras det? Hur erhålls erforderligt sekretesskydd?

Data förvaras som är gängse rutin i individuella CRF och hanteras enligt rutiner för sjukvårdens journaldata (låsta skåp, kontrollerad tillgång till data). Biobanksdata hanteras enligt biobankslagen som kräver spårbarhet och klartext möjlighet. Bilddata från fMRI hanteras med namnavkodande filer som löpnummermärkes. Alla magnetbaserade data hanteras med löpnummermärkning. Textbaserd data (loggbok, vissa svarsmallar) förvaras i klartext. Varje ingrepp och händelse journalförs i en loggpärm för varje deltagare. Sekretesskyddet hanteras genom att alla dokumnet med individidentifierande information sekretessbelägs och lämnas ut endast med sektretessförbehåll till vad myndigheten bedömer behöriga personer. Övriga data kan utlämnas utan att kränkning av individens intergitet kan uppkomma i den mening som anges i Helsingforsdeklarationen. Övervägandet att behålla textbaserde data i klartext är gjort utifrån att kontrollbarhet av data är lättare att garantera om insamlade data kan hanteras utan att anonymisering sker. Därmed kan varje datapunkt garanteras.

2:8 Redogör för tidigare erfarenheter (egna och/eller andras)

av den använda proceduren, tekniken eller behandlingen

Särskilt angeläget är att redovisning av risker för komplikationer görs tydliga och i förekommande fall med angivande av relevanta publikationer. Om ansökan avser fortsättning eller uppföljning av tidigare projekt, ange diarienummer samt datum för beslut av tidigare godkänd ansökan. Vid nya läkemedelsbehandlingar av patienter bör anges hur många patienter (med aktuell eller annan åkomma) som tidigare erhållit föreslagen eller högre dosering samt hur långa behandlingsperioder som studerats.

Den nödvändiga kompetensen tillhandahålls av Mats Lekander, expert om sambanden mellan immunfunktion, beteenden och hälsoperception (så kallat psykoneuroimmunologi); John Axelsson expert inom experimentell psykologi med inriktning inom sömn, kognition och hälsa; Caroline Olgart Höglund, expert inom neuroimmunologi, fysiologi och experimentell medicin; Martin Ingvar är expert inom hjärnavbildning och smärtreglering och överläkare vid MR Centrum där alla undersökaningar sker; Marie Lundberg är forskningssjuksköterska med lång erfarenhet.

Mats Lekander är medicine doktor, docent och sektionschef för Psykologisektionen vid Klinisk Neurovetenskap, Karolinska Institutet. Mats är specialiserad inom det tvärvetenskapliga området av psykologi, neurovetenskap och immunologi. Han har de senaste åren bland annat studerat samband mellan inflammationsmarkörer och självskattad hälsa (Lekander et al., 2004; Unden et al., 2007), men har även erfarenhet av hjärnavbildningsstudier som relaterar hjärnaktivitet med immunfunktion (Lekander et al., 2000).

Filosofie doktor John Axelsson har stor erfarenhet av att driva både fältstudier och experimentella studier om sambanden mellan neurobiologi och kognition som trötthet, prestation och välbefinnande (Axelsson et al., 2005; Axelsson et al., 1998; Axelsson et al., 2006; Axelsson et al., 2003; Axelsson et al., 2004; Nilsson et al., 2004). De senaste åren har John genomfört flera studier av hur störd sömn påverkar endokrinologiska funktioner, immunfunktion och självskatta hälsa (Axelsson et al., 2005; Axelsson et al., in preparation).

Prof. Martin Ingvar är chef för MR-centrum på Karolinska sedan 1998 och har ansvarat för en mängd fMRI-studier. Han leder en välfungerande forskargrupp som har god

kunskap om hur man planerar och genomför fMRI-studier. Forskargruppen har bland annat ett starkt fokus på kognition och smärtreglering.

Caroline Olgart Höglund är medicine doktor i fysiologi och docent i experimentell medicin vid Karolinska Institutet. Caroline leder ett forskartema inom neuroimmunologi vid Enheten för Lungmedicin, Inst för Medicin Solna och Inst för Fysiologi och Farmakologi. Hon har både framgångsrikt genomfört samt leder pågående humanstudier på friska försökspersoner samt alleriska patienter, inkl. astmatiker. Bl.a. har Caroline erfarenhet från komplicerade humanstudier där inverkan av psykologisk stress på immunfunktion studerats hos friska och allergiker. Caroline leder även flertalet djurexperimentella studier inom inflammations- och fysiologiområdet.

Endotoxininjektioner är en väl beprövad metod att aktivera en proinflammatorisk respons hos människor. Sammantaget har 140 studier genomförts sedan 70-talet med både friska, sjuka och äldre försökspersoner, varav vissa med uppemot 70 deltagare (Coyle 2006). I Sverige har 8 publicerade studier genomförts vilka inkluderat 87 injeceringtillfällen (Studier Soop et al. och Ungerstedt et al., 2000-2003).

Akutmedcinsk beredskap: Säkerheten på MR Centrum är som vid varje medicinsk klinik. Eftersom försöket innefattar injektion ingår att miinst en person med HLR A utbildning finns tillstädes. Akutlarmsfunktion med under en minuts inställelsetid för läkarbemannad hjärt lungräddning finns inom huset (Thoraxhuset som MR Centrum ligger vid).

3. Uppgifter om forskningspersoner

3:1 Hur görs urvalet av forskningspersoner

Med forskningsperson avses en levande människa som forskningen avser.

Ange urvalskriterier (inklusion och exklusion). På vilket sätt kommer forskaren i kontakt med/får kännedom om lämpliga forskningspersoner? Ange om rekrytering sker från (egna, andras) tidigare eller pågående studier. Om annonsering sker, skall annonsmaterialet insändas som bilaga. Om t.ex. barn, eller personer som tillfälligt eller permanent inte är kapabla att ge ett eget informerat samtycke skall tillfrågas om deltagande i projektet, skall detta särskilt motiveras. Om vissa grupper (t.ex. kvinnor, barn eller äldre) utesluts från deltagande i projektet skall detta särskilt motiveras.

Ett informationsblad (se bilaga) distribueras till studenter och anställda vid universitet och högskolor i Stockholm och hängs upp på campusområdet. Vi ämnar även hänga upp informationsmaterial på andra arbetsplatser vi får tillgång till och eventuellt göra en informationssida på internetsidan Facebook. 28 friska deltagare, hälften män och hälften kvinnor, kommer att gå igenom det slutliga protokollet. Inklusionskriterier är högerhänthet, fri från allergi, autoimmun sjukdom eller kronisk inflammation, god mental hälsa (frånvaro av klinisk depression och ångest), icke rökare, icke gravida, måttliga alkoholvanor, ej sömnstörningar eller övervikt (BMI <28) (båda är kopplade till en ökning av inflammationsmarkörer). Endast kvinnor som inte använder p-piller, och har en regelbunder menstruationscykel (± 2 dagar) kommer att delta. Kvinnor kommer att studeras under dag 3-11 i menstruationscykeln, under den follikulära fasen (mellan menstruation och ägglossning), för att kontrollera för variationer av immunfunktion under menstruationscykeln. Presumtiva deltagare kommer att genomgå en läkarundersökning med blodprov för att säkerställa exklusionsriterier.

3:2 Ange relationen mellan forskare/försöksledare och forskningspersonerna

☐ Behandlare (t.ex. läkare, psykolog, sjukgymnast) - forskningsperson (t.ex. patient, klient)

☐ Kursgivare (lärare) - student

☐ Arbetsgivare - anställd

☒ Annan relation. Beskriv: ingen

3:3 Redogör för det statistiska underlaget för studiepopulationens (ernas)/ undersökningsmaterialets(-ens) storlek

Redovisa en statistisk styrka, så kallad ”power”-beräkning eller motsvarande överväganden för tydliggörande av studiens möjligheter att besvara frågeställningarna.

28 deltagare (varav 14 kvinnor) genomgår båda betingelserna i en randomiserad ordning. Studien blir dubbelblind för endotoxin/placebo. Gällande endotoxinstimulering erhålls mycket starka effekter på immunparametrar och kognition; effektstorleken på immunsystemet är betydligt större än 3, vilket ger en power på 100%. Tidigare studier har visat på starka signifikanser med runt 10-20 deltagare på kognitiva test och med en effektstorlek på 0,75 har vi en power på över 99% i withinanalysen (placebo vs. endotoxin). Även för interaktionen har vi en mycket god power, över 97%. Anledningen till att ha 28 deltagare är att få tillräcklig power för fMRI-designen. Även om vi balanserar betingelserna (inom individer) måste man ha fler än 20 personer för att få fullgod power. En andra anledning för att använda 28 personer är att få tillräcklig power för att utvärdera könsskillnader. Här är svårt att uttala sig om exakta effektstorlekar då vi utvärderar kognitiva funktioner som inte tidigare studerats i fråga om könsskillnader med hjälp av MR-kamera.

Det är svårt att beräkna power in en analys som innehåller både ett within (endotoxin vs. placebo) och between (kvinnor vs. män) förfarande. En approximativ beräkning med hjälp av en ren withinanalys med 2 nivåer och en effektstorlek på 0.5 – 0.75 ger power på 96-100% för huvudeffekten och mellan 75 - 98% för interaktionen mellan kön och betingelse.

3:4 Kan forskningspersonerna komma att inkluderas i flera studier samtidigt eller i nära anslutning till denna studie

Ange om forskningspersonerna kan inkluderas samtidigt i flera studier eller i nära anslutning till denna studie. Ange i så fall projekttitel, forskningshuvudman, forskare som genomför studien (kontaktperson) samt diarienummer (om känt) för de övriga studierna. När avslutades ett eventuellt tidigare deltagande?

Nej, inget planerat men inget hinder finns

3:5 Vilket försäkringsskydd finns för de forskningspersoner som deltar i projektet

Det åligger forskningshuvudmannen att kontrollera att befintliga försäkringar täcker eventuella skador som kan uppkomma.

Projektet ingår i klinisk forskning som är del av ordinarie verksamhet vid MR Centrum och därför ingår försökspersonerna i patientförsäkringen vilket anses tillfyllest.

3:6 Vilken ekonomisk ersättning eller andra förmåner utgår till de forskningspersoner som deltar i projektet och när betalas ersättningen ut (Utförligare beskrivning kan lämnas i bilaga)

Ersättning för obehag och besvär. Belopp (före skatt):	4000	
Ersättning för förlorad arbetsinkomst	☐ Ja	☒ Nej
Reseersättning	☒ Ja	☐ Nej
Befrielse från kostnader för läkemedel	☐ Ja	☒ Nej
Befrielse från andra kostnader. Vilka?		
Andra förmåner. Vilka?		
När betalas ersättningen ut?	efter genomfört deltagande	
Ingen ersättning betalas ut	☐	

4. Information och samtycke

4:1 Proceduren för och innehållet i den *information* som lämnas då forskningspersoner tillfrågas om deltagande

Beskriv hur och när information ges och vad den innehåller. Vem informerar? Normalt skall en kortfattad och lättförståelig skriftlig information ges. Denna skriftliga information skall bifogas ansökan. Om ingen eller ofullständig information ges, måste skälen för detta noggrant anges.

Forskningspersonen skall informeras om

- den övergripande planen för forskningen
- syftet med forskningen
- de metoder som kommer att användas
- de följder och risker som forskningen kan medföra
- vem som är forskningshuvudman och kontaktperson
- att deltagandet i forskningen är frivilligt och
- forskningspersonens rätt att när som helst avbryta sin medverkan.

Ett kortfattat informationsblad (bilaga 4:1 och 4:3) ges till intresserade deltagare. De personer som visat fortsatt intresse att delta i studien, kontaktas för en utförlig beskrivning av studien. Den muntliga informationen stämmer väl överens med den skriftliga, men ger mer utrymme för frågor och förtydliganden (se bilaga 4:2).

4:2 Hur och från vem inhämtas *samtycke*

Beskriv proceduren; vem som frågar, när detta sker och hur samtycket dokumenteras. Utförlig redovisning är särskilt viktig då barn eller personer med nedsatt beslutskompetens ingår i studien, likaså vid studier av en grupp (grupper), t.ex. föreningar, organisationer, företag, kyrkosamfund och församlingar eller arbetet i en skolklass.

Presumtiva deltagare kommer att informeras om samtycke i samband med att de träffar försöksledaren första gången. Därefter kommer deltagaren att kontakta försöksledaren om han/hon har bestämt sig för att delta. Då kommer deltagaren även att bokas in för en läkarundersökning. Vid samma tillfälle kommer försöksledaren att gå igenom protokollet ytterligare en gång. Anonymitet betonas och att individuella resultat inte kommer att redovisas. Alla deltagarna är väl informerade om att de kan avsluta studien när de vill utan närmare förklaring. Deltagaren kommer att lämna ett skriftligt samtycke där deltagaren skriver under på att han/hon är medveten om att han/hon kan avbryta försöket när som helst utan att meddela varför.

5. Forskningsetiska överväganden

5:1 Redogör för risker som deltagandet kan medföra samt möjliga komplikationer

Detta kan vara t.ex. smärta, obehag eller integritetsintrång som projektet innebär eller kan innebära.
Har åtgärder vidtagits för att förebygga de risker som sägs ovan? Vilken beredskap finns att hantera dessa komplikationer? Ange metoder som kommer att användas för att efterforska, registrera och rapportera oönskade händelser.

De viktigaste etiska aspekterna gäller om det är säkert och berättigat att utsätta försökspersoner för två fMRI-undersökningar och en endotoxininjektion (vid det ena experimenttillfället ges endast placebo).

Deltagandet i fMRI-studier är en säker och väletablerad metod som utförs av erfaren personal vid MR-centrum, KS. Försökspersonen informeras om hur MR-kameran ser ut och låter i förväg, och om någon upplever obehag i små utrymmen kommer personen avrådas från att delta i experimentet. Temperatur och puls registreras kontinuerligt.

Deltagaren kommer enligt praxis bära öronskydd inne i kameran. Under hela försöket kan försökspersonen kommunicera med försöksledaren, och han/hon kommer att hålla en alarmknapp i handen som kan användas om deltagare önskar avbryta MR-scanningen omedelbart av någon anledning.

Endotoxininjektion är en etablerad modell för experimentell immunaktivering både i djur och hos människa. Endotoxinstimulering ger influensaliknande symptom, som feber, frysningar, ökad hjärtfrekvens, huvudvärk och muskelsmärtor. Dessa symptom kan motverkas med en tablett med ibuprofen (400 mg) om försökspersonen önskar det, och studien avbryts då. Experimentet utförs på sjukhusområdet (KS) under läkares observation, och injektionen handhas av sjukhuspersonal med tidigare erfarenhet av liknande studier. Ett visst obehag kan upplevas under isättandet av venkatetern, och detta lindras genom att lokabedövande kräm appliceras i armvecket 10 minuter i förväg.

Ytterligare obehag kan upplevas under smärtperceptionstestet (värme mot handrygg). Även detta test har använts av forskargruppen tidigare och orsakar endast måttligt obehag. Smärtan är kortvarig och upphör omedelbart efter försökets avslut, och ger inga bestående skador eller brännmärken. Även under detta test har deltagaren möjlighet att säga stopp när som helst.

Vi anser inte att de formulär som deltagarna ska fylla i är integritetskränkande eller utlämnande (se bifogade bilagor), men vi ger försökspersonen en chans att ta ställning till detta i och med första mötet med försöksledaren, 3-4 veckor innan experimentet, då försöksledaren går igenom vilka formulär som kommer att användas. Samtliga frågor har används i tidigare studier.

5:2 Redogör för förutsebar nytta för de forskningspersoner som ingår i projektet

Deltagarna kommer att kunna få tillgång till sina egna data vid önskemål och ersätts ekonomiskt för deltagande (4000). Vi ser inga andra fördelar med deltagandet.

5:3 Gör en egen värdering av förhållandet risk – nytta för de forskningspersoner som deltar

Även om lättare sjukdomsbesvär troligtvis kommer att uppstå så är de lindriga och kortvariga vid den låga dos endotoxin som används - de minskar redan efter 5-6 timmar och är helt borta inom 24 timmar. Tidigare studier visar på minimala effekter på sömn om stimuleringen sker så tidigt på dagen. Nyttan för försökspersonen själv är en bättre kunskap om sina egna biologiska och kognitiva reaktioner vid sjukdom. Vid intresse får naturligtvis deltagarna även tillgång till huvudresultaten via artiklar och rapporter efter att dessa är publicerade (de som anmäler intresse får mail om detta vid publicering).

5:4 Identifiera och precisera vilka etiska problem t.ex. risk – nytta i ett vidare perspektiv som kan uppstå inom eller genom projektet

De etiska problem som föreligger rör primärt deltagarnas obehag under endotoxinstimulering, testning i MR-kameran, och under smärttestet. I ett vidare perspektiv är de etiska problemen små, då riskerna för försökspersonerna är mycket små, och nyttan av kunskap runt sjukdomsmekanismer, och beteende och upplevelse under sjukdom är stor. En trolig utveckling är att vi utför fler studier med liknande protokoll där vi studerar olika sjukdomstillstånd eller riskgrupper, exempelvis allergiker,för att jämföra effekten av en endogen och kronisk immunaktivering med experimentiell och kontrollerad aktivering som i den gällande studien. Sammantaget bedömer vi att nytta med undersökningen överstiger det obehag försökspersonerna utsätts för.

6. Redovisning av resultaten

6:1 Hur garanteras forskningshuvudmannen och medverkande forskare tillgång till data (anges vid t.ex. uppdragsforskning) och vem ansvarar för databearbetning och rapportskrivning

Vid uppdragsforskning anges hur forskningshuvudmannen och medverkande forskare garanteras tillgång till data och vem som ansvarar för databearbetning och rapportskrivning.

Ansvaret för studien ligger inom Karolinska Institutet. Det medicinska ansvaret är samlat under verksamhetschefen vid MR Centrum. Eventuella samarbetspartners under

försökets gång får tillgång till data och manus före publicering. Ingen extern vetorätt för publikation. Martin Ingvars forskargrupp kommer att ansvara för statistikbearbetning av fMRI-ananlyser och kognition. Mats Lekanders grupp kommer att ansvara för analyser av immunparametrar och skattningar.

6:2 Hur kommer resultaten att göras offentligt tillgängliga
Kommer studien att insändas för publicering i tidskrift eller publiceras på annat sätt

Ange i vilken form resultaten planeras offentliggöras samt tidsplan för detta.

Resultaten kommer att publiceras i vetenskapliga tidsskrifter. De kommer även att redovisas vid internationella kongresser, utbildning samt vid andra presentationer. Det är även troligt att resultaten kommer att ingå i bokkapitel och intervjuer, då flera av forskarna regelbundet förekommer i media.

6.3 På vilket sätt garanteras forskningspersonernas rätt till integritet när materialet offentliggörs/publiceras

Beskriv procedurer eller metod för avidentifiering/anonymisering. Redovisas endast resultat på statistisk gruppnivå?

Redovisning av resultat sker endast på statiskisk nivå.

7. Redovisning av ekonomiska förhållanden och beroendeförhållanden

7:1 Vid uppdragsforskning

Ange uppdragsgivaren t.ex. vid klinisk läkemedelsprövning.

Namn: Kontaktperson:

Adress: Telefon/mobiltelefon:

Ange uppdragsgivarens relation till forskningshuvudmannen/medverkande forskare, t.ex. anställningsförhållande.

7:2 Redovisa eventuella ekonomiska överenskommelser med uppdragsgivare eller andra finansiärer (namn, belopp)

Vid klinisk läkemedelsprövning kan hänvisning ske till ingånget avtal med sjukvårdshuvudmannen eller genom uppgift om föreslagen ersättning enligt överenskommelsen mellan Landstingsförbundet och LIF som bifogas. Separata överenskommelser med den/de som skall genomföra forskningen skall också redovisas. Om överenskommelserna inte är klara i sin helhet vid tidpunkten för ansökan skall belopp för studien/ersättning till kliniken/genomföraren och vad ersättningen skall täcka alternativt belopp per forskningsperson anges här. Vid studier där fler än en forskningshuvudman deltar skall principerna för och storleksordningen för ersättning för studien i sin helhet anges.

Uppdragsgivarna (ett flertal forskningsstiftelser) har inget annat förhållande till forskarna är att de beviljat medel för studien.

7:3 Redovisa forskningshuvudmannens och medverkande forskares intressen/tillgångar

Redovisa de som kan tänkas påverka tilltron till objektiviteten i genomförande och rapportering (t.ex. aktieinnehav eller konsultuppdrag i finansierande företag).

Inga andra intressen andra än rent forskningsmässiga föreligger.

8. Förteckning över bilagor

Dokument som, i tillämpliga fall, skall bifogas om inte motsvarande information finns i blanketten har markerats med x. Markera de bilagor som skickas in med denna ansökan.

Insänd med	Bil	Beskrivning	Klinisk	Annan

ansökan	nr		läkemedels prövning	forskning
☒	1<br>p 1:5	Deltagande forskningshuvudmän och medverkande forskare (kontaktpersoner) vid forskning där mer än en forskningshuvudman deltar	x	x
☒	2<br>p 2:1	För fackmän avsedd projekt/forskningsplan (protokoll), vid behov även för lekmän avsedd bilaga	x	x
☒	3<br>p 3:1	Annonsmaterial för rekrytering av forskningspersoner	x	x
☒	4<br>p 4:1	Skriftlig information till dem som tillfrågas	x	x
☒	5<br>p 2:4	Enkät, frågeformulär	x	x
☐	6	Gemensam EU-blankett (gäller fr.o.m. den 1 maj 2004), gäller även vid ändring	x	
☐	7	Sammanfattning av protokollet på svenska	x	
☐	8	Prövarhandbok alt. bipacksedel/produktresumé	x	
☐	9<br>p 2:6	Intyg från verksamhetschef/motsv. om resurser	x	x
☒	10	CV för forskare (samma som p 1:3) med huvudansvar för genomförande (redovisa forskarens kompetens)	x	x
☐	11<br>p 3:6	Beskrivning av ersättning till forskningspersoner	x	x
☐	12<br>p 7:1<br>p 7:2	Överenskommelser med uppdragsgivare/finansiär om t.ex. anställningsförhållanden, bidrag/ersättning till prövningsplats, sjukvårdshuvudman, forskningshuvudman eller forskare	x	x
☐	13	Tillstånd från strålskyddskommitté eller motsvarande	x	x

9. Undertecknande

Behörig företrädare för sökande forskningshuvudman enligt p 1:2.

Ort: Datum:

Signatur

Namnförtydligande:

Undertecknad forskare som genomför projektet (kontaktperson) enligt p 1:3 intygar härmed att forskningen kommer att genomföras i enlighet med ansökan.

Ort: Datum:

Signatur

Namnförtydligande:

Anvisning för ansökan

Denna ansökningsblankett används vid ansökan om etikprövning enligt lagen (2003:460) om etikprövning av forskning som avser människor. Den är avsedd att användas för all slags forskning där godkännande skall inhämtas från en etikprövningsnämnd. Blanketten skall användas även vid begäran om rådgivande yttrande enligt 2 § förordning (2003:616) med instruktion för regionala etikprövningsnämnder. Beroende på vilken forskning som ansökan gäller kommer de uppgifter som efterfrågas nedan att ha olika relevans. Därmed

varierar också kravet på utförlighet i redovisningen av dessa. Markera på formuläret när uppgiften inte berör det aktuella projektet. Ansökan skall ifyllas så att den blir lättläst, dvs. den skall inte vara handskriven och inte skriven med liten stil och kort radavstånd. Blanketten skall skrivas på svenska.

Observera att en ansökan aldrig är komplett (och därmed kan behandlas) förrän blanketten är korrekt ifylld och avgiften är betald.
Ansökan med bilagor insändes i ett original och 16 kopior!

Om denna blankett
Allt som skall fyllas i är markerat grått. Övrig förklarande text är låst och får inte ändras. De grå fälten expanderar allteftersom du skriver, det finns alltså obegränsat utrymme för din text. Vid utskrift syns enbart texten du fyllt i och ej de grå fälten som syns på skärmen, det är därför viktigt att kontrollera att allt verkligen är ifyllt. När du är klar skriver du ut blanketten, kompletterar med erforderliga underskrifter samt bilagor och skickar till den regionala etikprövningsnämnd till vars upptagningsområde forskningshuvudmannen hör, se www.forskningsetikprovning.se.
