# Supplementary material for: The effect of a transient immune activation on subjective health perception in two placebo controlled randomised experiments
Source: PLoS One. 2019 Mar 6;14(3):e0212313. doi: 10.1371/journal.pone.0212313 (PMC6402640; doi:10.1371/journal.pone.0212313)
Supplement: S3 File — (PDF) [file pone.0212313.s004.pdf]

Regionala etikprövningsnämnden i Stockholm

2009 -07- 29

Dnr: 2009 / 1273-32

Handl: [illegible]

Ansökan om godkännande av tillägg till studien: "Inflammation och Hjärnfunktion"

(Dnr. 2008/955-31)

a) Vi önskar ha möjlighet utföra genetiska test på insamlade blodprover vid ett senare tillfälle. Av intresse är genetiska variationer (t ex. COMT-genen) relaterade till inflammation, affekt och smärta. Bifogad är den godkännande- och informationsblankett som försökspersonen får skriva under gällande biobank, genetiska tester och PUL. Ändringar som gjorts i försökspersonsinformationen i den tidigare, redan godkända ansökan, har markerats med gult (Bilaga 1, 2, 3).

b) I den tidigare ansökan godkändes mätning av hur utseende och rörelsemönster påverkas vid en akut inflammation respons. Vi önskar även mäta kroppsdoften enligt följande protokoll:

Kroppslukten kan förmedla information om släktskap, sinnesstämning och diet. Anekdotiskt stöd finns också för att kroppslukten kan ha ett diagnostiskt värde som markör för olika sjukdomar. Med denna backgrund vill vi testa hypotesen att kroppslukten kan vara en markör för den inflammatoriska respons som följer på injektion av endotoxin.

Vi mäter deltagarnas kroppslukt genom att de strax före injektion får sätta på sig en kroppsnära t-tröja med amningsskydd insydda i armhålan. Deltagarna får på ett tidigt stadium innan de kommer för testning instruktioner om hur de ska förhålla sig til faktorer som påverkar kroppslukten (rökning, kryddad mat, deodorant, luktande produkter, etc.). Tröjan bär tröjan i 5-timmar. Därefter förvaras skydden i luktfria fryspåsar under -30 °C och tinas upp inför ett senare test med andra deltagare där skydden utgör luktstimuli. Femtio personer kommer delta i detta senare test. Deltagarna bedömer luktstyrka, attraktivitet, behaglighet, och "sjuklighet" hos luktstimuli från både endotoxin- och kontrollgruppen. Beräknad tidsåtgång för deltagaren är totalt 5min för att sätta på och ta av tröjan.

c) Vi har i tidigare ansökan angivit att vi kommer fästa en venkateter i armvecket på försökspersonen. Vi önskar ändra detta till att ha en kateter i vartdera armvecket. Genom den ena venkatetern injiceras endotoxinet (placebo), och den andra används för dragning av blod. På så sätt undviker vi kontamination av blodproverna då spår av

endotoxin kan fastna på insidan av venkatetern. En van sjuksköterska kommer att applicera venkatetrarna. Beräknad tidsåtgång är 10 minuter för att sätta i en extra venkateter.

d) Ett huvudsyfte med studien är att studera om akuta inflammationsresponser påverkar smärtreglering. Vi har etiskt tillstånd att mäta hur endotoxin påverkar smärtkänslighet för värmesmärta (mätt på underarmen). Vi ämnar tillägga mätning om endotoxin har effekt på smärtkänslighet för trycksmärta (tryck på tumnageln, tidsåtgång 20min). Vi ämnar även studera om endotoxin påverkar smärttrösklar för värme och trycksmärta (tidsåtgång 5+5min), samt smärtförstärkning/hämning. Det senare, var mekanism kallas DNIC (diffuse noxious inhibitroy control), kommer att kombinera ett ”cold pressor” test (deltagaren har handen i 4°C vatten så länge den kan upp till max 1min) samtidigt som deltagaren genomför ett smärtkänslighetstest (tryck på tumnageln). Detta test mäter förstärkning/försvagning av smärtimpulser (tidsåtgång 5*1min). Alla dessa test är välvaliderade och har genomförts i Martin Ingvars forskargrupp. Deltagarna kommer att tydligt informeras att de kan avbryta testet när de vill. Dessa test kommer att användas i pilotstudien om 8 personer (se deltagarinformation för denna pilotstudie (Bilaga 4) med gulmarkerade ändringar). Endast de smärttest som visar på god effekt av endotoxin hos majoriteten av deltagarna i pilotstudien kommer att användas i huvudstudien där deltagarna kommer att genomföra testen i MR-kamera.

Syftet med att addera flera smärttest i pilotstudien är således att öka specificiteten att finna eventuella effekter av endotoxin på smärta samt även minska risken att genomföra onödiga smärttest i huvudstudien. Den sammanlagda testtiden kommer att var något förlängd i pilotstudien jämfört med ursprungsansökan (20minuter). Att detta inte ökar med mer beror på att vi inte kommer genomföra testet social exklusion som fanns med i grundansökan.

[signature] -- Sth 090626 --

Mats Lekander Plats, datum

Huvudansvarig forskare för projektet

GODKÄNNES Dat. 2009-08-27

Pierre Lafolie [signature]
Regionala etikprövningsnämnden
i Stockholm

EPN erinrar om skyldighet till förhandsprövning av Datainspektionen vid

Kopians överensstämmelse med originalet intygas:

[signature]

Ann-Christin Becker
Administrativ sekreterare
