# Supplementary material for: The effect of a transient immune activation on subjective health perception in two placebo controlled randomised experiments
Source: PLoS One. 2019 Mar 6;14(3):e0212313. doi: 10.1371/journal.pone.0212313 (PMC6402640; doi:10.1371/journal.pone.0212313)
Supplement: S4 File — (PDF) [file pone.0212313.s005.pdf]

<u>Ansökan om godkännande av tillägg till studien: "Inflammation och Hjärnfunktion"</u>

Vi har fått etiskt godkännande för studien "Inflammation och Hjärnfunktion" (Dnr. 2008 / 955-31) med inompersondesign, samt godkännande av ett tillägg för att mäta smärtkänslighet under immunaktivering (Dnr. 2009 / 1273-32). I denna ansökan önskar vi ändra studiedesignen till mellangruppsdesign, samt lägga till två smärtkänslighetstest.

Vi har nu genomfört en pilotstudie med 8 deltagare med endotoxinstimulering, men utan fMRI-scanning, i dubbelblind cross-over design enligt vårt tidigare etiska tillstånd. Det blev i denna testomgång tydligt att vi inte kan hålla studien blindad för inompersonsjämförelser med endotoxin vs placebo. Effekten av dosen var helt enligt förväntan och motsvarade symptomen för en begynnande influensa och gick över inom ca. 3 timmar. Deltagarna fick inte feber och blodtryck och hjärtfrekvens påverkades inte. Då tydliga influensasymptom uppstår i ca. hälften av deltagarna kunde vi inte kontrollera för förväntanseffekter när försökspersonerna fått endotoxin, och reagerat tydligt på det, vid första tillfället. Det var för samtliga försökspersoner efter andra omgången tydligt vid vilken omgång de fått endotoxin, då vi hade ett längre samtal angående hur de upplevt studien, om de känt sig trygga, om informationen varit tillräcklig och om vår "sjukdomsmodell" varit för krävande eller obehaglig.

För att kunna genomföra studien på ett så rättvisande sätt som möjligt önskar vi därför göra några ändringar i studieprotokollet.

A) Vi tror att blindningen av studiebetingelserna inte är fullständig och att de kognitiva konsekvenserna av detta är svåra att hantera och kan störa studien. Sannolikt kan smärttesten i hög grad påverkas av förväntanseffekter vid de båda tillfällena. Vi önskar behålla dosen som den är, då den ger önskad effekt, men vill göra en **mellangruppsdesign** istället för som tidigare en inomgruppsdesign. **Vi ansöker därför om etiskt godkännande för att ta in dubbelt så många försökspersoner, 56 personer totalt, men att varje person endast deltar vid ett tillfälle. Ersättningen blir 1500 kronor.** Hälften av försökspersonerna får endotoxin, andra hälften saltlösning. Vi kommer att informera alla om att de får endotoxin på samma sätt som tidigare, och behandla varje testtillfälle som om vi injicerat endotoxin. Som tidigare kommer endast den ansvariga läkaren veta vad som injicieras, och forskningsledaren kommer som tidigare ha minimal kontakt med läkaren. Alla andra som arbetar kring försökspersonen kommer vara blinda för betingelsen.

Regionala etikprövningsnämnden i Stockholm
2010 -08- 27
Dnr: 2010/1352-32
Handl:

Ett huvudsyfte med studien är att studera om akuta inflammationsresponsen påverkar smärtreglering. Som väntat skilde sig de olika smärttesten i pilotstudien åt något men överlag har vi fynd som tyder på en påverkan av endotoxin. Som vi skrivit tidigare kommer endast de smärttest som visar på god effekt av endotoxin hos majoriteten av deltagarna i pilotstudien att användas i huvudstudien.

B) Smärta är - definitionsmässigt - en subjektiv upplevelse. Våra försökspersoner svarar bl.a. med visuell analog skala (VAS) och vi frågar alltid uttryckligen om individens subjektiva upplevelse här och nu. I detta sammanhang är det dock även önskvärt med mer objektiva mått. Ett välvaliderat sådant är benets så kallade flexionsreflex - LLFR (Lower Limb Flexion Reflex). Detta är ett neurofysiologiskt mått som erbjuder ett fönster mot spinala (dvs. ryggmärgs-) och supraspinala (dvs. hjärnstams och hjärn-) mekanismer för smärtreglering, dvs. processer som vi intresserar oss för i det aktuella försöket. Enligt EFNS-(European Federation of Neurological Societies)-guidelines är LLFR den mest välanvända av samtliga nociceptiva reflexer och förefaller vara den mest tillförlitliga för att utvärdera behandling av smärta [1].

Hur går test av Lower Limb Flexion Reflex till?

Två för ändamålet avsedda ytelektroder anbringas mot den avtvättade huden ovanför nervus suralis på utsidan av ena foten. Dessa kopplas m h a säkerhetskoppling till Biopac-enheten STMISOL, avsedd och godkänd för humanexperiment för denna och liknande applikationer. Systemet har en rad säkerhetsfunktioner och är givetvis fullständigt isolerad från yttre strömkällor. Varje stimulering består av en följd av ett fåtal millisekunder långa rektangulära pulsar med en frekvens på 100-300 Hz. Ytsensorer (2st + en jord) för elektromyografisk (EMG) mätning av reflexen klistras på nedre delen av lårmuskulaturen (m biceps femoris). En enkel titrering, utgående från en ström på 0 mA och med en ökning om mellan ca 2 och 4 mA per steg, görs enligt en vanligt förekommande step-wise algoritm för att fastställa den aktuella försökspersonens tröskel för utlösande av reflexen. Efter detta ges ett antal övertröskliga stimuleringar enligt gängse metodik och muskelsvar mäts med EMG.

Det blir aktuellt med en mätning före respektive efter endotoxin/kontroll injektion. Vi har upparbetat en god erfarenhet av denna metod inom Prof. Martin Ingvars grupp, där lovande resultat med denna teknik från en nyligen avslutat studie håller på att sammanställas. Den totala tiden för varje smärttest blir ca. 30 minuter.

C) Det finns djurexperimentella data som talar för att TNF-α underhållen inflammation bidrar till uppkomsten av neuropatisk smärta [2]. En föreslagen modell som till viss del efterliknar den brännande känsla som är en del av sådan smärta, är Thunbergs illusion (även kallad Thermal Grill Illusion). Illusionen uppkommer när i sig inte smärtsamma varma (ca. 42 °C) respektive svala (ca. 16 °C) element läggs omlott och upplevs som ett lätt brännande obehag. Det har föreslagits att Thunbergs illusion är en lämplig modell för vissa aspekter av neuropatisk smärta [3]. Den brännande förnimmelsen är fördragbar, säker och momentant reversibel då man drar undan handen/underarmen från termoden. Graden av detta obehag skattas t.ex. med VAS. Att använda en enkel skattning av smärta med denna illusion inom den aktuella modellen för inflammation kan öka förståelsen kring de processer som sker på spinal och kortikal nivå i samspelet mellan smärta och inflammation. Vi har inom gruppen arbetat upp en god kompetens kring denna typ av testning och har en för ändamålet lämplig termod tillverkad av ett svenskt medicintekniskt företag. Testet sker efter i övrigt avslutat huvudförsök. Testet sker under den tid då försökspersonen ändå är kvar i sjukhuslokalen för postexperimentell övervakning.

Sammanfattningsvis ansöker vi om etiskt tillstånd för följande förändringar i den aktuella studien:

A) Mellangruppsdesign ersätter inomgruppsdesign så att blindning blir möjlig och vi undviker förväntanseffekter som kan påverka resultatet.

B) Lower limb flexion test läggs till i syfte att mäta smärtresponser på ett mer objektivt sätt.

C) Thermal grill illusion test läggs till för att undersöka om akut immunaktivering har någon effekt på system relevanta för neuropatisk smärta.

Referenser:

1. Sandrini, G., et al., *The lower limb flexion reflex in humans.* Prog Neurobiol, 2005. **77**(6): p. 353-95.
2. Leung, L. and C.M. Cahill, *TNF-alpha and neuropathic pain--a review.* J Neuroinflammation. **7**: p. 27.
3. Craig, A.D., *Editorial: Can the basis for central neuropathic pain be identified by using a thermal grill?* Pain, 2008. **135**: p. 215-216.

[signature]

Stockholm, 100823

-- --

Mats Lekander, docent, projektansvarig

Plats, datum

GODKÄNNES Dat. 2010 -09- 14

[signature]

Hans Glaumann
Regionala etikprövningsnämnden
i Stockholm

Kopians överensstämmelse med originalet intygas:

[signature]

Anne Manninen
