# Supplementary material for: The effect of a transient immune activation on subjective health perception in two placebo controlled randomised experiments
Source: PLoS One. 2019 Mar 6;14(3):e0212313. doi: 10.1371/journal.pone.0212313 (PMC6402640; doi:10.1371/journal.pone.0212313)
Supplement: S5 File — (PDF) [file pone.0212313.s006.pdf]

APPLICATION FOR ETHICAL VETTING

For information concerning the application: ***see Appendix and Guidance,*** ***(www.epn.se)***

IT IS NOT POSSIBLE TO USE THE ENGLISH VERSION OF THE APPLICATION.

IT IS NOT PERMITTED TO FILL IN THE APPLICATION IN ENGLISH, ONLY SWEDISH.

To the Regional Ethical Review Board in:

State the Regional Ethical Review Board to which the entity principally responsible for the research belongs. For a list of boards see *(www.epn.se)*

Date fee paid:

Please note that an application is never complete (and thus able to be processed) until the form is correctly filled in and the fee has been paid.

Project title:

Give a descriptive title in Swedish for laymen, without using confidential information.
Where suitable, also state the identity of the project and the number, date, version etc of the project/research plan (protocol or testing plan).

Information to be completed by the Regional Ethical Review Board

Application complete:	Case number:
Request for additional information concerning the application:	Requested information received:
Date of decision:	Date processed:

The application concerns (also applies when an advisory statement is requested):

research in which only one responsible research body participates (5 000 kr)

research in which more than one responsible research body participates (16 000 kr)

research in which more than one responsible research body participates,
but in which all the researchers or subjects of research have an immediate
link to only one of the responsible research bodies (5 000 kr)

merely processing personal data (5 000 kr)

research involving clinical trials of medicinal products (16 000 kr)

changes to a previously approved application in accordance with section 4 of Statute (2003:615) concerning the Ethical Review of Research Involving Humans (2 000 kr)

If the board decides that the legislation concerning ethical review is not applicable to the study/research project, is an advisory statement wanted?

Yes: No:

1. Information concerning the entity principally responsible for the research etc.

1:1 The entity principally responsible for the research

The application for ethical vetting of research is to be made by the entity principally responsible for the research. By entity principally responsible for the research is meant a government authority or a physical or legal entity under whose auspices the research is conducted. Research within the state is primarily conducted at the seats of learning, but also at certain other authorities, such as the National Council for Crime Prevention and the National Board of Health and Welfare. Municipalities and county councils can also be those principally responsible for the research, as can legal entitles according to civil private law

Name:

Address:

1:2 Qualified representative for the entity principally responsible for the research

This is a qualified representative of the entity principally responsible for the research (e.g. head of department, head of unit, head of operations). The entities principally responsible for the research are themselves to decide upon by internal consultation, delegation, or by means of power of attorney, the responsible research body which is qualified to act as a representative. A copy of the document in question must be submitted.

Name: Professional title:

Address:

1:3 Researchers who are primarily responsible for the completion of the project (principal contact)

Name: Professional title:

Address:

E-mail:

Telephone:

Mobile phone:

1:4 Other participants

Other persons principally responsible for the research, plus researchers responsible for completing the project locally (principal contacts) are to be listed here or in an annex stating names and addresses (see p. 9, annex 1).

1:5 Account for the access to the resources needed during the implementation of the project

State which person or persons are responsible (head of department, head of operations or the equivalent) for the safety of those participating in the research at all the units/clinics where the persons are participating. Affidavits from those responsible *must* be appended (see p. 9 annex 9). It must be clear from the affidavit that all the necessary financial, structural and human resources are available to guarantee the safety of those participating in the research .

1:6 Applications/notifications to other authorities

Clinical testing of medicinal products

For an application for a permit from the Swedish Medical Products Agency: www.mpa.se

Application submitted (date) Permit granted

EudraCT number:

Cases concerning some kinds of genetic research

If personal data concerning genetic dispositions that have been revealed as a result of a genetic examination is to be dealt with in the study, this must be notified to the Swedish Data Inspection Board in accordance with section 10 of the Personal Data Ordinance (1998:1191). See the Data Inspection Board's home page: www.datainspektionen.se

Notification submitted (date) Will be submitted once approval is granted after ethical vetting

Certain research involving the irradiation of research subjects

The application, in accordance with sections 16 and 22 of the regulations of the Swedish Radiation Safety Authority (SSMFS 2008:35) concerning general obligations with respect to medical and odontological activities involving ionizing radiation, is to be submitted to the Radiation Protection Committee. For more information, contact the relevant local radiation protection committee.

Application submitted (date): Application confirmed

2. Information concerning the project

2:1 Summary of the research project (programme)

The description must be comprehensible to all board members . It is therefore advisable to avoid specialist terminology. State the background and the purpose of the study, together with the scientific question(s) for which answers are being sought. State the most important variables affecting the investigation. State what advances in knowledge can be expected as a result of the project and what the significance of these might be. State if it is a study of records, if the research is an assignment, etc. Detailed information in the research plan, protocol or programme that is intended for specialists must be appended as an annex (see p. 9, annex 2). A more detailed description *that is intended for laymen* concerning the implementation of the study can, if needed, be appended to the obligatory research plan intended for experts.

2:2 What is/are the primary scientific question(s) forming the basis of the design of the project?

If the project can be described as testing a hypothesis, state the primary hypothesis and the secondary hypothesis if there is one. Referral can be made to more detailed information for experts in the form of an appended research plan in accordance with 2:1

2:3 State the results from relevant animal experiments

If animal experiments have not been carried out, the reasons for this must be given.

2:4 Give an overview of the examination procedures used, data collection and the nature of the data.

It should be clear from the description, how it is planned to complete the project. Describe the nature of data that has been collected. Describe how the reliability of the data is ensured (e.g. quality control/monitoring). When questionnaires and interviews are used, the procedure used should be described and, for example, the content of questions and how conclusions are drawn. Questionnaires and rating scales <u>must</u> be appended (see p. 9 annex 5). For medical research should be stated, for example, the types of intervention, the methods of measurement, the number of visits, the time required each time research is carried out and the means used to administer any pharmaceutical preparation and/or isotopes and the amount of blood samples taken (including the accumulated amount when multiple testing takes place). State also if the examination procedure etc differs from routine clinical measures and if so, in what way. State which procedure can be required to administer the prospective treatment after the conclusion of the study. State the procedures used to collect biological material. Account for data sources and procedures when processing personal date. For more detailed information referral can be made to the appended research plan.

2:5 Describe how biological material that has been collected is to be stored in a biobank

By a biobank is meant is meant biological material that has been collected and retained from one or more persons, the origins of which can be traced to the person or persons from which it had originated and is being kept pending further notice or for a certain period of time.

Describe how each sample that is to be retained is to be kept, the coding procedures and what conditions apply when material is released. State who is the person responsible for the biobank. Note that in appropriate cases the biobank is to be notified to the National Swedish Board of Health and Welfare in accordance with the Biobanks in Medical Care Act (2002:297)

2:6 Record-keeping, registering and processing of data

State how the examination procedures and any operations that take place are to be recorded. Account for how the registration and processing of the results will take place. If the material is to be coded, state the procedure: who will be keeping the code list in safe custody and which person or persons have access to them; where they are being kept and for how long and also if the material will be rendered anonymous or destroyed. State if audio and video recordings are to be used. Describe how accessible the data material is, how it is kept and how the requisite confidentiality is attained.

2:7 Describe previous experience (your own and/or others) of the procedure, technique or treatment used.

It is particularly important that the risks of complications are clearly accounted for and that the relevant publications are listed. When patients are to be given a new form of treatment, such as a pharmaceutical product, it should be stated how many patients (with the complaint in question or another one), had previously been given the proposed treatment, dosage of a pharmaceutical product (or a different dosage) and over what period of time the treatment has been studied.

3. Information about the participants in the research

3:1 How are participants in the research chosen?

By participant in the research is meant a living person who is the subject of the research. State the selection criteria (inclusion and exclusion). Give an account of the manner in which the researcher will get in contact with/become aware of suitable participants for the research. State if recruitment will take place from your own studies, the previous studies of others or ongoing studies. If advertising is to take place, the advertising material *must* be submitted as an annex (see p. 9 annex 3). If, for example, children or people who, temporarily or permanently, are not capable of themselves giving their informed consent are to participate in the project, this is to be specifically justified. If certain groups (women, children or the elderly, for example) are to be excluded from the project, this is to be specifically justified.

3:2 State the relationship between the researchers/the leader of the research and those people participating in the research

Person administering the treatment (e.g. doctor, psychologist, physiotherapist) - person participating in the research (e.g. patient, client)

Course facilitator (teacher) - student

Employer - employee

Any other relationship that could possibly entail a risk of influence. Describe:

3:3 State the statistical foundation with respect to the size of the population(s) and/or material(s) studied

Give an account of the statistical power, the so-called power-calculation, or give an account of equivalent considerations which clarify the study's ability to answer the questions posed.

3:4 State if participants in the research may be included in several studies, either simultaneously or in another study or other studies closely linked to this one. If so, what kind of research?

3:5 What insurance cover is there for research participants taking part in the project?

It is the responsibility of the entity principally responsible for the research to check that existing insurance policies cover any injuries that may arise.

3:6 What financial remuneration or other benefits are participants in the research entitled to and when is this to be paid? (A more detailed description can be submitted as an annex)

Compensation for discomfort and inconvenience. Sum (before tax):

Compensation for income from employment	Yes	No
Allowance for travelling expenses	Yes	No
Exemption from costs of pharmaceutical products	Yes	No

Exemption from other costs Which?

Other benefits Which?

When is compensation paid?

No compensation to be paid

4. Information and consent

4:1 The procedure involved and the content of the information that is given when subjects are asked to participate in the research

Describe how and when the information is given and what it contains. Indicate who provides the information. Brief and easily comprehensible written information should normally be given. This written information must be attached to the application (see p. 9 annex 4). If no information or incomplete information is given, detailed reasons for this must be given.

4:2 How is consent to be obtained and from whom?

Describe the procedure, who asks, when this takes place and how the consent is documented. Exhaustive documentation is particularly important, not only when children or people with a diminished ability to make decisions participate in the study of a group or groups such as associations, organisations, companies, church communions, congregations, or school classes.

5. Considerations in the light of research ethics

5:1 Describe the risks that participation might entail and possible complications

This could mean, for example, physical injury, pain, discomfort or other violations of personal integrity that the project entails or can entail. State not only which measures been taken to prevent the risks mentioned above, but also what preparations have been made to deal with these complications. Give an account of the methods that will be used to investigate, register and report undesirable events.

5:2 Describe the predictable benefit for the people participating in the research who are part of the project

5:3 In a broader perspective, identify and specify which ethical problems, such as risk versus benefit, can arise as a part of or as a result of the project

Here, for example, an account can be given of which groups can be identified or given help as a result of the study.

6. Presenting the results

6:1 How are both the entity principally responsible for the research and research collaborators guaranteed access to data (to be stated when the research is an assignment) and who is responsible for processing data and writing reports?

6:2 How will the results be made publicly available? Will the study be sent for publishing in a journal or published in some other manner?

State the format in which it is planned to make the results public and indicate the time frame that is applicable.

6:3 In what manner will the right to integrity of those participating in the research be guaranteed when the material is made public or is published?

Will only results at a statistical group level be shown? Describe the procedures or the methods used for concealing identities or ensuring anonymity.

7. Reporting the financial circumstances and dependencies

The purpose of the accounts to be given in points 7:1 - 7:3 is to clarify all direct or indirect circumstances that could possibly affect the researcher's relationship to the persons participating in the research (during the process of information, consent or implementation, for example).

7:1 When the research is an assignment

State who the principal is: for example a company (when there is clinical testing of pharmaceuticals or the testing of other new products, for example) an organisation or an authority.

Name: Principal contact:

Address: Telephone/mobile phone:

State the relationship between the entity principally responsible for the research/participating researchers and the principal assigning the research (employee/employer for example).

7:2 Give an account of any financial agreements with a principal or any other financiers (Name, amount)

Where the clinical testing of pharmaceuticals is concerned, reference should be made to agreements entered into with the principal responsible for health care. Similar agreements may occur when other research is commissioned and is to be accounted for in the same way. Separate agreements with the person or persons who are to complete the study are also to be presented. State the amount that is allocated for the study/compensation to the clinic/person completing the research and what the compensation is to cover. Any sums given to persons participating in the research should also be accounted for here (see p. 9, annex 12).

7:3 Give an account of the interests of the entity principally responsible for the research, the principal researcher and of participating researchers

An account is to be given here of, for example, ownership of shares, employment status, consultancy work for companies providing finance and any companies owned by researchers that could benefit financially, directly or indirectly, as a result of the research (see p. 9, annex 12).

8. Signatories

Authorized representative for the entity principally responsible for the research who is the applicant in accordance with p. 1:2.

Place: Date:

Signature: ______________________________

Clarification of signature:

Official title:

The undersigned researcher who is carrying out the project (principal contact) in accordance with p.1:3 hereby certifies that the research will be carried out in accordance with the application.

Place: Date:

Signature: ______________________________

Clarification of signature:

Official title:

9. List of annexes

Documents which, in appropriate cases, are to be appended if corresponding information is not on the form, are marked with an x. Mark those annexes that are to be submitted with this application.

Send with application	Annex nr	Description	Clinical testing of pharmaceuticals	Other research
	1	The participating entity principally responsible for the research and collaborating researchers (principal contacts) for research involving the participation of more than one entity principally responsible for the research. For information see p. 1:5	x	x
	2	Research plan intended for specialists. Also an annex intended for laymen, if needed. For information see p. 2:1 and Guidance to the research plan/protocol (programme).	x	x
	3	Advertising material for the recruitment of research participants For information see p. 3:1 and Guidance to the application p. 3:1.	x	x
	4	Written information for those who have been asked. For information see p. 4:1 and Information for research participants.	x	x
	5	Questionnaire. For information see p. 2:4	x	x
	6	Standard EU form (as of 1 May 2004). Also applicable when changes are made	x	
	7	Summary of the protocol in Swedish	x	
	8	User's handbook or product insert/product summary/IB	x	
	9	Testimonial from operations manager or equivalent concerning resources and the safety of those participating in the research. For information see p. 2:6.	x	x
	10	CV of researcher (same as p. 1:3) with primary responsibility for completion (give an account of the competence of the researcher(s) that is of relevance to the study. Information in Guidance to the application p. 1:3.	x	x
	11	Description of remuneration given to research participants. For information see p 3:6 and Guidance to the application p. 3:6	x	x
	12	Agreements with principals/financiers concerning, for example, terms of employment, grants/compensation awarded to the place where the research is conducted, to the principal responsible for heath care, to the entity principally responsible for the research or to the researcher. For information see p. 7:2 and 7:3	x	x

Other annexes appended to the application:
