# Supplementary material for: The effect of a transient immune activation on subjective health perception in two placebo controlled randomised experiments
Source: PLoS One. 2019 Mar 6;14(3):e0212313. doi: 10.1371/journal.pone.0212313 (PMC6402640; doi:10.1371/journal.pone.0212313)
Supplement: S6 File — (DOCX) [file pone.0212313.s007.docx]

2.1

Background

Recognition by immune system cells of foreign microorganisms triggers the release of hormone-like signalling agents known as proinflammatory cytokines, which have the capacity to activate an inflammatory process. In addition to the peripheral inflammatory reaction, they also affect the central nervous system (CNS), where a high concentration of cytokine receptors is present within structures that have an important regulatory function, such as in the hypothalamus. Animal experiments have shown that stimulation of such receptors results in several pronounced changes in behaviour, known as sickness behaviours.

General activation of the immune system is associated with fatigue, increased sleepiness, changes in food preferences, social withdrawal, increased sensitivity to pain and memory impairment, which taken together are referred to as the disease response. These behavioural changes are reminiscent of the nonspecific and diagnostically challenging symptoms that are commonly presented in primary care and include fatigue, depression and pain sensitivity. Despite high clinical relevance, strikingly little experimental research has been conducted on human subjects regarding the effects of peripheral inflammation on the brain. The studies that have been carried out on humans have focused on how activation of the immune system affects memory and learning, sleep and appetite, and to some degree, symptoms of anxiety. The sole human study that has explored how the experimentally activated immune system affects brain activity found that activity in the insular cortex, an area located between the parietal and temporal lobes and which is responsible for registering changes in the inner environment of the body, and in the frontal portion of the anterior cingulate cortex (ACC) correlate with reduced pulmonary function and a higher load of proinflammatory cytokines in asthmatics who were provoked with an allergen. The study indicates that these portions of the brain are important to the subjective experience of disease and discomfort that occurs when the immune system is activated, such as during inflammation.

We have shown in several cross-sectional studies a strong correlation between inflammatory markers (cytokines) and subjective perception of health. The research literature shows that the cytokine-induced disease response entails profound changes in behaviour, which suggests a shift in attention from external (perceptive) to internal (interoceptive, e.g. pain and itching) signals. Studies also show a link between inflammation and pain. Research has shown that both interoceptive perception and perception of pain are associated with activity in the insular cortex and ACC. For this reason, we would like to explore how experimental activation of the immune system using endotoxin (an established model for systemic inflammation) affects the subjective experience of ill-health, and whether attention in humans shifts towards internal signals under the influence of immune system activation. We want to investigate how pain sensitivity is affected, since we believe that activity in specific areas of the brain will be affected by the immune response – more precisely, the insular cortex and ACC. We believe that such activation of the brain is one of the mechanisms underlying sickness behaviour.

In conjunction with this study, a pilot study will be conducted to investigate changes in appearance and patterns of movement during illness. We will have both doctors and non-professionals study videos and photographs of the gait and facial expressions of the subjects to determine whether the disease response can be visually interpreted. Please see the project description for details.

Purpose and scientific questions

The purpose of the study is to explore how activation of the immune system affects brain activity (primarily ACC and insular cortex), cognition (perception of external versus internal stimuli and pain) and subjective perception of health. We would further like to assess whether there is a correlation between the degree of immune activation and the strength of the impact on brain activity and cognition, as well as whether gender differences may be present.

Key study variables

Blood samples will be drawn for analyses of peripheral immune activation (proinflammatory cytokines such as TNF-alpha, IL-1β and IL-6, as well as anti-inflammatory activity involving IL-1ra). The participants will be asked to assess their perceived health, mood and level of fatigue in conjunction with blood sampling and fMRI examination. In the first cognitive test, which concerns perception of external versus internal stimuli, the subject will assess whether the presented audio signals (10 tones) coincide with the subject’s heartbeat, and whether they include a change in pitch. The second cognitive test will measure whether and how immune activation affects pain sensitivity, using heat stimulation applied to the back of the hand. The third cognitive test will assess whether sickness behaviour affects how subjects react to social exclusion, which can be considered to be a type of “social” pain. The participant will engage in a virtual game with two fictitious partners and will at times be excluded from the game. The subjects will also be photographed and videotaped on one occasion during the experiment, for later use of photos and videos in the pilot study.

Advances in knowledge and importance of results

Diffuse symptoms (fatigue, depression, increased pain sensitivity, changes in appetite) are extremely common; rather than being disease-specific, they appear to be common to many conditions of ill-health and may in part be related to a low-grade inflammatory effect on the brain. Since such symptoms are determinants for self-rated health, which is an important marker of ill-health as well as a risk factor for sick leave, a better understanding of how the immune system affects brain function, symptoms and behaviour is of great interest. As a whole, the study will generate knowledge about the mechanisms underlying general symptoms of illness. The purpose of the pilot study is to generate knowledge about the processes that affect how the health of an individual is perceived by external observers, primarily medical personnel. Such knowledge is highly relevant for clinical diagnostics.

2.2

Our primary hypothesis is that immune activation helps to reset the focus of attention from external to internal signals that results in increased processing of interoceptive (heartbeat) versus perceptual (pitch)

information. Similarly, we believe that activation of the immune system increases sensitivity to both pain and social exclusion. We also anticipate that activation of the immune system will result in temporary perception of a worsening in subjective health. We believe that the mechanism underlying these anticipated effects of immune activation relates to increased activity in the relevant areas of the brain, primarily the insular cortex and ACC.

2.3

Animal studies have shown that behaviour and brain function are affected by experimental peripheral immune stimulation, and also by direct administration of cytokines in the central nervous system. Animals treated with endotoxins (bacterial waste products recognised by the immune system) show signs of depressive behaviour and sickness behaviour such as withdrawal, decreased social interaction, reduced appetite, decreased exploratory behaviour and elevated sensitivity to pain. Stimulation has been shown to activate structures such as the amygdala and hypothalamus. Animal studies have significant limitations because behaviour may be difficult to interpret and transfer to humans, and because mild immune activation becomes difficult to evaluate since behavioural changes in animals are often quite subtle. For this reason, substantially higher endotoxin doses are used in animal studies than in studies on humans. This study is primarily concerned with the subjective human experience of disease – a question that is impossible to answer through animal experiments.

2.4

Each subject will participate in the study on two separate occasions during which the same protocol will be repeated – once under the influence of endotoxin and once using placebo (PBS). Prospective participants will be screened (screening questionnaire appendix 5:1) 3–4 weeks prior to participation, at which time an initial visit with the doctor is scheduled. During the visit to the doctor a blood sample will be drawn to check C-reactive protein (CRP) and blood count, especially leukocytes (5 ml). Elevated CRP levels would suggest ongoing infection and inflammatory activity in the body, in which case the individual would be ineligible to participate in the study. This test will also be repeated on the morning of the study. Complete blood count with differential assessment of leukocytes shows the number of white blood cells the individual has and the percent of each type present in the blood. Subjects should demonstrate normal levels. Ten to 14 days prior to participation, the study leader will review the protocol with the subject and explain the self-rating scales. Participants will also receive a 4-day sleep/health diary (appendix 5:2) in which they will begin to record data 2 days prior to the FMRI measurements. Participants will be instructed to sleep 7–8 hours (within the 10 p.m. to 8 a.m. time frame) two nights before the fMRI to avoid the effects of sleep deficit and disruption of circadian rhythm on cognition and immunological parameters (behavioural guidelines appendix 4:3).

Participants will arise in the morning between 6:30 and 8:00 a.m. on the same day that the tests are to be conducted. A CRP sample will be drawn (5 ml) at 10:00 a.m. at the MRI Centre and the study leader will review the health diary with the subject. 10.00 at MRI Centre and the study leader will go through the health diary with the subject. Should the CRP be elevated or if the subject notes in the health diary an upper respiratory infection or other illness over the preceding 2 days, the study sampling will be postponed and the individual will return at a later date. Participants will be instructed to eat lunch between 12 noon and 1:30 p.m. and to arrive at the MRI Centre at 1:45 p.m. Most of the protocol will be carried out with the participants lying down. At 2:15 p.m. an intravenous (i.v.) catheter will be placed on the inside bend of the elbow. At 3:30 p.m. the first blood sample will be drawn. Five minutes later the subjects will be injected with either endotoxin (0.8 ng/kg LPS from E. coli) or placebo. Subsequently blood samples will be drawn through the venous catheter at 4:30, 5:30 and 6:30 p.m., as well as after completion of the fMRI protocol at 7:30 p.m. Each blood sample (5) over the course of the study will remove 17 ml of blood, which will subsequently be analysed for cytokine and hormone levels. Thus a total of 90 ml of blood will be removed that day (and a total of 185 ml during the entire study). Plasma samples will be stored at -70 °C for further analysis. Preparations for the fMRI study will be initiated at 5:45 p.m. once the camera becomes available, since it is used for clinical purposes during the daytime. The subjects may drink water throughout the course of the study and will receive a light meal on two occasions.

Laboratory protocol (overview)

10:00 a.m. Blood sample for CRP analysis (ensure eligibility for participation). Meeting with study leader and review of health diary. (20 min)

1:45 p.m. Subject arrives lab, removes outerwear (time required 30 min)

2:15 p.m. Subject lies down and is prepared for i.v. catheter placement (15 min)

2:30 p.m. Health self-rating for baseline (questionnaire 5:3) (5 min)

2:35 p.m. Rest, light meal (approx. 55 min)

3:30 p.m. Blood sample and health self-rating for baseline (questionnaire 5:3) (5 min)

3:35 p.m. Injection with endotoxin/placebo (5 min)

3:40 p.m. Rest (50 min)

4:30 p.m. Blood sample and health self-rating (questionnaire 5:3) (5 min)

4:35 p.m. Rest (55 min)

5:30 p.m. Blood sample and health self-rating (questionnaire 5:3) (5 min)

5:35 p.m. Photographing and filming gait (10 min)

5:45 p.m. Preparations fMRI protocol (15 min)

6:00 p.m. fMRI – baseline (20 min)

6:20 p.m. Tone/heart rate part 1 (12 min)

6:32 p.m. Rest and blood sample (5 min)

6:37 p.m. Tone/heart rate part 2 (12 min)

6:49 p.m. Rest and health self-rating (questionnaire 5:4) (5 min)

6:54 p.m. Pain perception (12 min)

7:06 p.m. Rest and health self-rating (questionnaire 5:3) (5 min)

7:11 p.m. Social exclusion part 1 (6 min)

7:17 p.m. Rest (questionnaire 5:3) (5 min)

7:22 p.m. Social exclusion part 2 (6 min)

7:28 p.m. Blood sample and health self-rating (questionnaire 5:4) (5 min)

7:33 p.m. Emotional self-rating of faces (7 min)

7.40 p.m. Exit fMRI, snack, health self-rating (questionnaire 5:3)

Endotoxin stimulation

Endotoxins consist of bacterial cell wall lipopolysaccharides (LPS) that activate the immune system, but do not cause bacterial infection. For the purposes of this study we have chosen LPS from the E. coli serotype O113, which has been used in human studies, especially in the US, since 1976 and also in Sweden in recent years by Sollevi and Soops’ research group at KH (Ref. no. 416/98, 240/99, 269/99, 377/02, 436/03, 467/4, 1305/31). Endotoxin studies approved in Sweden have used higher doses (1.0 – 4.0 ng/kg), but we plan to use a dose of 0.8 ng/kg since our interest lies in eliciting a mild immune response that will lead to subtle subjective experiences, and we wish to avoid strong physical reactions in our study subjects. Such lower doses have previously been used in both cognitive and sleep studies. The cognitive studies primarily used endotoxin from Salmonella bacteria in which the maximum cognitive effects occurred about 3 hours after injection. Because different types of endotoxins, and even different production batches, may vary somewhat in their effect, we would like to conduct a small preliminary study in which the maximum cognitive and subjective effect is achieved specifically using our endotoxin to allow us to later optimise our protocol using ± 1–0.5 hour time intervals (see section 3:1 for more information). Our endotoxin will be imported from Prof. Suffredini’s laboratory at the NIH since he was instrumental in developing and testing this specific type of endotoxin in humans (Suffredini et al. 1999). The endotoxin is produced according to Good Laboratory Practice (GLP) guidelines and transported in powder form (see the Drug Fact Sheet appendix for details about this reference endotoxin). After mixing and dilution, the infusion is sterilised by autoclaving prior to injection and is free of bacteria and fungi.

Experimental endotoxin stimulation results in influenza-like symptoms such as fever, chills, increased heart rate, nausea, headache and muscle pain. The usual experimental dose in human studies is 2–4 ng/ml. Lower doses (0.2–0.8 ng/kg) do not affect body temperature and heart rate to the same extent, but some malaise and fatigue may be present 2–6 hours following injection, and resolve completely within 24 hours. The symptoms can be eliminated if needed using ibuprofen, which can be administered at the request of the participants, but in such cases the experiment must be discontinued because the medicine affects the parameters under study.

Blood sampling and assessment of inflammatory markers

An intravenous catheter will be placed 60 minutes prior to endotoxin stimulation, and a blood sample will be taken each hour throughout the protocol beginning 5 minutes before endotoxin stimulation. The blood samples will be immediately analysed at the conclusion of the protocol for reasons related to the white cell blood count (differential). The plasma will be frozen at -70 °C and analysed after the conclusion of the study for inflammatory markers (pro-inflammatory markers such as TNF-alpha, IL-1β and IL-6, anti-inflammatory activity (IL-1ra)) and hormones (cortisol). The concentration of these substances in the blood will be measured using enzyme-linked immunosorbent assay (ELISA) methodology, which has a sensitivity that covers both normal levels and strongly stimulated (elevated) levels of cytokines/hormones.

Assessments (appendix 5:1–5:4)

In conjunction with the blood samples and following the cognitive tests using fMRI, participants will assess their health, as well as symptoms such as fatigue, sleepiness, appetite, anxiety, depression etc. The participants will also fill in a sleep/health diary each morning and evening (amount of time required 5 minutes) for four days. We will primarily use questions selected from previously developed and well-validated scales to measure anxiety, depression and stress (Spielberger State-Trait Anxiety Inventory=STAI and Hospital Anxiety and Depression=HAD), fatigue (Swedish Occupational Fatigue Inventory=SOFI), appetite (Spiegel et al., 2004) and sleepiness (Karolinska Sleepiness Scale=KSS). In addition, we will use 2 scales to measure self-rated health, one for overall health (Self-rated health=SRH-5) and one to measure perception of health over the past hour (7-point Likert scale, Axelsson et al. in prep). The health scales are based on different time lines.

fMRI protocol

The first cognitive test involves testing of either 1) an assessment of whether or not the stimuli (10 tones) are timed to coincide with the heartbeat, *or* 2) whether or not a change in pitch occurs during any of the 10 tones. The two tests will be carried out separately and in alternating order. Our approach will be based on the original protocol obtained from Prof. Critchley at University College in London, and published in Nature Neuroscience (Critchley et al., 2004). The test measures the extent to which the attention of the individual is directed towards either internal or external signals.

The second cognitive test will measure pain sensitivity using a Medoc-TSA 2001 heat analyser. A 2x3 cm metal probe will be used to heat various points on the back of the hand over a period of 12 seconds. The temperature varies between 40 and 49 degrees Celsius and the subject will rate the temperatures on a Visual Analogue Scale (VAS) where 0 indicates “no heat at all” and 10 indicates “greatest tolerable pain”. A calculation will be made of what temperature is experienced as level 5 on the VAS scale for each individual, and using statistical analysis we will see whether the 50% sensitivity boundaries change during endotoxin stimulation.

The third cognitive test will measure the perception of social exclusion, which is a measure of “social pain”. Subjects will play a computerised ball game that is displayed on the fMRI screen, and are told that they are each playing with two other people who are sitting in different rooms. What they are actually playing is a preprogrammed interactive ball game. Over the course of the game the two other players will gradually exclude the subject from the game, and this is perceived as being excluded. Studies show that the perceived discomfort is associated with neural activation in the ACC, and that this activation pattern of the brain overlaps to some extent with what occurs during physical pain.

We expect to see changes in the ACC and insular cortex, which are both involved in introception and perception of pain. In summary, these cognitive tests have been successfully used in previous fMRI studies. We have access to original programs and protocols, all of which have been well tested.

A short assessment (7 min) of another person’s emotional status will also be carried out; the person will assess the photos of faces with various emotional expressions. The contrast between assessing what emotional expression a person has and whether I like the person tests the cortical control mechanism of emotional activation in the limbic system (Nyberg/Öhman, in submission).

We will also carry out a small preliminary study to assess the quality of procedures and establish the final protocol. One test session (5–8 subjects) will be carried out in which the subject is stimulated with endotoxin and then answers all questionnaires and does the tests, but does not participate in the MRI, to see the exact amount of time required for the questionnaire and to ensure that the quantity of questions is manageable for the subject. In this part of the preliminary study we also want to determine the optimal timing for maximum sickness response, with a precision of 1–0.5 hours. Since no prior endotoxin studies have primarily focused on sickness response and the subjective experience, it is important to determine that this will optimally coincide with the MRI insofar as possible. A second session (5–8 subjects) will be carried out including the MRI, but without endotoxin stimulation, to determine the exact timing of the fMRI protocol.

2.5

A code key will be created and a code number assigned to each participant. All data collection will be recorded with a code number and will not be traceable to the participant without access to the code key. The only exception would be blood count and CRP samples, which will be turned in to the KH chemistry lab for immediate analysis, where the same procedures will be used as in clinical practice (personal identity number). Plasma from all blood samples drawn on the afternoon of the day of the experiment will be stored frozen at -70 °C on the Karolinska Hospital campus and labelled with code number, category (A/B) and time of sampling. The blood test results will be stored under lock and key at the Psychology section, Clinical Neuroscience. They will then be coded according to the code key. The code key will not be stored on a computer, but rather as a hard copy stored under lock and key. A CD copy will also be created and placed under fireproof storage. No samples will be released to unauthorised individuals. Upon request, individuals will be provided with access to their own test results.

2.6

The study will be carried out within the framework of grants and R&D funding. The necessary resources will be provided by the Department of Clinical Neuroscience and the MRI Centre.

2.7

Data will be stored according to standard procedure in individual CRFs and handled in accordance with medical record procedures (locked cabinets, controlled access to data). Biobank data will be handled in accordance with the Swedish Biobanks Act, which requires traceability and plain text. Imaging data from the fMRI will be handled in sequentially numbered anonymised files. All magnetic-based data will be sequentially numbered. Text-based data (log, certain response templates) will be stored in plain text. Each procedure and event will be recorded in a log binder for each participant. Confidentiality will be maintained by keeping all documents with individual identifying information confidential, and only disclosed, subject to confidentiality, to individuals whom the authority considers to be authorised. The remainder of the data may be disclosed without violating the privacy of the individual as defined by the Helsinki Declaration. The decision to retain text-based data in plain text was taken because it is easier to verify data if the relevant data can be processed without anonymisation. In this way, each data point can be verified.

2.8

The necessary experitse are provided by Mats Lekander, expert on the relationship between immune function, behaviour and health perception (known as psychoneuroimmunology); John Axelsson, expert in experimental psychology with a focus on sleep, cognition and health; Caroline Olgart Höglund, expert in neuroimmunology, physiology and experimental medicine; Martin Ingvar is an expert in brain imaging and pain control and a senior physician at the MRI Centre where all investigations are conducted; Marie Lundberg is a research nurse with extensive experience.

Mats Lekander holds an MD-PhD and is an associate professor and head of the Psychology Section at the Department of Clinical Neuroscience, Karolinska Institutet. Mats specialises in the interdisciplinary fields of psychology, neuroscience and immunology. His studies over the past few years have included inflammatory markers and self-rated health (Lekander et al., 2004; Unden et al., 2007), and he has also been involved in brain imaging studies that relate brain activity to immune function (Lekander et al., 2000).

John Axelsson, PhD, has extensive experience directing both field studies and experimental studies regarding associations between neurobiology and cognition, including fatigue, performance and well-being (Axelsson et. al., 2005; Axelsson et. al., 1998; Axelsson et. al., 2006; Axelsson et. al., 2003; Axelsson et. al., 2004; Nilsson et al., 2004). In recent years John has conducted several studies on how disturbed sleep affects endocrine function and immune function, as well as self-rated health (Axelsson et al., 2005; Axelsson et al., in preparation).

Prof. Prof. Martin Ingvar has been head of the MRI Centre at Karolinska since 1998 and has been responsible for many fMRI studies. He heads an well-run research group with expertise in planning and conducting fMRI studies. His research group is strongly focused on cognition and pain regulation.

Caroline Olgart Höglund holds a PhD in physiology and is an associate professor of experimental medicine at Karolinska Institutet. Caroline heads a research team in neuroimmunology at the Respiratory Medicine Division, Dept. of Medicine, Solna, and the Dept. of Physiology and Pharmacology. She has successfully conducted human studies and is currently leading a study on healthy subjects and allergy patients, incl. asthmatics. Caroline has experience from complicated human studies in which the impact of psychological stress on immune function was studied in both healthy subjects and allergy patients. Caroline also heads up several animal studies concerning inflammation and physiology.

Endotoxin injections are a well-tested method of provoking an inflammatory response in humans. In all, 140 studies have been carried out since the 1970s on healthy, sick and older subjects, some of which included as many as 70 participants (Coyle 2006). In Sweden, eight published studies have been carried out, which included a total of 87 occasions where injections were administered (Studier Soop et al. och Ungerstedt et al., 2000–2003).

Emergency medical preparedness: Safety at the MRI Centre meets safety standards commensurate with medical facilities. Since this experiment involves injections, at least one person with ACLS training is present. An emergency alarm system with a doctor response time for cardiopulmonary resuscitation of less than one minute is present in-house (Thoracic Surgery Building in which the MRI Centre is located).

3.1

An information leaflet (see appendix) will be distributed to students and employees at universities and university colleges in Stockholm and posted on the campuses. We also intend to post information material at other workplaces to which we have access and possibly create a Facebook page with information. In all, 28 healthy subjects, half men and half women, will complete the final protocol. Inclusion criteria are right-handedness; no allergies, autoimmune disease, or chronic inflammation; good mental health (absence of clinical depression and anxiety), nonsmokers, nonpregnant, moderate alcohol consumption, no sleep disturbances or overweight (BMI <28) (both are linked to an increase in inflammatory markers). Only women who do not take oral contraceptives and who have regular menstrual cycles (± 2 days) will be eligible to participate. Women will be studied during days 3–11 of their menstrual cycle, during the follicular phase (between menstruation and ovulation), to assess for variations in immune function during the menstrual cycle. Prospective participants will undergo a physical examination including drawing blood samples to ensure absence of exclusion criteria.

3.2

No study leader – subject relationships

3.3

In all, 28 participants (including 14 women) will follow through with both categories in random order. The study will be double-blinded for endotoxin/placebo. Concerning endotoxin stimulation, extremely strong effects will be obtained on immunological parameters and cognition; effect size on the immune system is considerably greater than 3, which yields a power of 100%. Earlier studies have shown high significance with around 10–20 participants in the cognitive test; an effect size of 0.75 will yield a power >99% in the “within” analysis (placebo vs. endotoxin). Interaction is also associated with high power, >97%. The reason for having 28 participants is to achieve sufficient power for the fMRI design. Although we are balancing the categories (within individuals) more than 20 subjects are needed to achieve adequate power. A second reason for including 28 subjects is to achieve sufficient power to assess for gender differences. It is difficult to express an opinion concerning exact effect sizes since we are evaluating cognitive functions that have not previously been studied in regard to gender differences using an MRI.

It is difficult to calculate power in an analysis that includes both a “within” (endotoxin vs. placebo) and a “between” (women vs. men) approach. An approximate calculation using a pure “within” analysis across 2 levels and an effect size of 0.5–0.75 yields a power of 96–100% for the principal effect and between 75–98% for the interaction between gender and category.

3.4

No, no current plans but no obstacles either

3.5

The project falls under clinical research which is part of regular services at the MRI Centre and therefore the subjects are covered by patient insurance, which is considered to be adequate.

4.1

A brief information leaflet (appendices 4:1 and 4:3) will be provided to prospective participants. Those who continue to show interest in participating in the study will be contacted for a detailed description of the study. The oral information is highly consistent with the written information, but provides greater opportunity for questions and clarifications (see appendix 4:2).

4.2

Prospective participants will be informed about consent during their first meeting with the study leader, after which the prospective participants will get back to the study leader concerning their decision on participation. At that time the participant will also be scheduled for a physical examination with the doctor. At the same time the study leader will review the protocol once again. Anonymity and nondisclosure of individual results will be emphasised. All participants will be carefully informed that they may withdraw from the study at any time without further explanation. Participants will provide signed written consent in which they state that they are aware that they may withdraw from the study at any time without providing an explanation.

5.1

The most important ethical considerations concern whether it is safe and justifiable to expose subjects to two fMRI examinations and one endotoxin injection (on one of the experimental occasions, only placebo will be administered).

Participation in fMRI studies is a safe and well-established method which is conducted by experienced personnel at the KH MRI Centre. Subjects will be informed ahead of time about the appearance of the MRI equipment and the associated sound volume; anyone who experiences discomfort in small spaces will be dissuaded from participating in the experiment. Temperature and pulse will be continually recorded. Participants will wear hearing protection inside the MRI machine according to standard procedure. Throughout the entire experiment, subjects will be able to communicate with the study leader and will hold an alarm button in their hand that can be used if participants wish to terminate the MRI immediately for any reason.

Endotoxin injection is an established model for experimental immune activation in both animals and humans. Endotoxin stimulation results in influenza-like symptoms such as fever, chills, increased heart rate, headache and muscle pain. These symptoms may be alleviated with an ibuprofen tablet (400 mg) should the subject so desire, in which case the study will be terminated. The experiment will be conducted at the KH campus under a doctor’s supervision, and the injection will be handled by hospital personnel with prior experience of similar studies. Some discomfort may be experienced when placing the venous catheter, and this will be mitigated by applying local anaesthetic cream to the cubital fossa (inside of the bend of the elbow) 10 minutes beforehand.

Additional discomfort may be experienced during the pain perception test (application of heat to the back of the hand). This test has also been used by the research group previously and causes only moderate discomfort. The pain is limited in duration and immediately ceases after the conclusion of the experiment; it does not cause any permanent injuries or burn marks. Participants will also have the opportunity to say stop at any time during this test.

We do not believe that the questionnaires that participants will fill out intrude on privacy or that they disclose sensitive information (see enclosed appendices), but we give subjects the opportunity to express their opinions on this during the first meeting with the study leader, 3–4 weeks prior to the experiment, at which time the study leader will review the questionnaires that will be used. All questions have been used in previous studies.

5.2

Participants will be able to access their own data upon request and will receive financial compensation for their participation (SEK 4000). We see no other benefits associated with participation.

5.3

Even though some symptoms are likely to occur, they are mild and short-lived at the low dose of endotoxin that is to be used – they will begin to decrease after just 5–6 hours and completely resolve within 24 hours. Prior studies show minimal effects on sleep when stimulation occurs so early in the day. The benefit for subjects personally is improved understanding of their own biological and cognitive reactions to disease. Participants who are interested will of course have access to the main findings through articles and reports once they are published (those who express interest will receive an email about this upon publication).

5.4

Existing ethical problems primarily concern the discomfort to participants during endotoxin stimulation, testing during the MRI examination, and during the pain test. From a broader perspective, the ethical problems are minor, since the risks to subjects are extremely small, while the benefit from knowledge regarding disease mechanisms, behaviour and experience during disease is great. A likely development is that we will conduct more studies using similar protocols in which we will study various disease conditions or risk groups, such as allergy patients, to compare the effects of endogenic and chronic immune activation with experimental and controlled activation such as in the study at hand. All things considered, we believe that the benefit of the study outweighs the discomfort to which subjects are exposed.

6.1

Karolinska Institutet is responsible for the study. The clinical administrator of the MRI Centre holds the aggregate medical responsibility for the study. Partners, if any, over the course of the study will have access to data and the manuscript prior to publication. No one will have external rights to veto publication. Martin Ingvar’s research group will be responsible for statistics processing of the fMRI analyses and cognition. Mats Lekander’s group will be responsible for analysing immunological parameters and evaluation of self-rated health.

6.2

The results will be published in scientific journals. They will also be presented at international congresses, for educational purposes and in other presentations. It is also likely that the results will be included as chapters in books and in interviews, since several of the researchers are regular media contributors.

6.3

The results will be presented only from the standpoint of statistics.

7.1

NA

7.2

The project was commissioned by a number of research foundations whose sole relationship with the researchers is the funding granted for the study.

7.3

No interests other than purely research-related interests are involved.
