# Supplementary material for: The effect of a transient immune activation on subjective health perception in two placebo controlled randomised experiments
Source: PLoS One. 2019 Mar 6;14(3):e0212313. doi: 10.1371/journal.pone.0212313 (PMC6402640; doi:10.1371/journal.pone.0212313)
Supplement: S7 File — (DOCX) [file pone.0212313.s008.docx]

Application for approval of addition to the study: “Inflammation and Brain Function”

(Ref. no. 2008/955-31)

1. The previously approved application entailed a pilot study to investigate how appearance and patterns of movement are affected during illness, and we would like to conduct an additional pilot study to address body odour as described below:

Body odour can convey information regarding relationships, mood and diet. Anecdotal data also suggest that body odour may be of diagnostic value as a marker for various diseases. Given this situation, we would like to test the hypothesis that body odour can act as a marker for the inflammatory response to injection of endotoxin.

We will sample the body odour of participants by having them put on a tight-fitting t-shirt with nursing pads sewn into the armpits immediately prior to injection. Participants will receive instructions at an early stage prior to arrival for testing concerning how they should act in relation to various factors that affect body odour (smoking, spicy food, scented products etc.). The participants will return the shirts following the fMRI session after having worn them for about 5 hours. The nursing pads will be stored at -30 °C in odour-free freezer bags and later defrosted prior to subsequent tests with other participants where the pads will serve as sources of olfactory stimulation. Fifty subjects will participate in this latter test. The participants will assess odour strength, how pleasant or unpleasant it is, and whether there is a sense of “illness” in the odours from the stimuli provided by both the endotoxin and control groups.

1. Moreover, we would like to have the opportunity to conduct genetic testing on the blood samples at a later time. Our interest is potential genetic variations related to inflammation. Enclosed is the consent and information form that subjects will sign regarding biobank, genetic testing and the Personal Data Act. Changes made to the information provided to subjects in the previously approved application are highlighted in yellow.
2. In the previous application we stated that we will place a venous catheter in the cubital fossa (inside of the bend of the elbow) of the subject. We would like to change this to placement of a catheter in each cubital fossa. One will be used for injecting endotoxin (placebo), and the other for drawing blood. By doing so we will avoid contaminating blood samples since traces of endotoxin may adhere to the inside of the catheter.
