# Supplementary material for: The effect of a transient immune activation on subjective health perception in two placebo controlled randomised experiments
Source: PLoS One. 2019 Mar 6;14(3):e0212313. doi: 10.1371/journal.pone.0212313 (PMC6402640; doi:10.1371/journal.pone.0212313)
Supplement: S8 File — (DOCX) [file pone.0212313.s009.docx]

Application for approval of addition to the study: “Inflammation and Brain Function”

We have received ethical approval for the study “Inflammation and Brain function” (Ref. no. 2008 / 955-31) with within-person study design, as well as approval for an add-on to measure pain sensitivity during immune activation (Ref. no. 2009 / 1273-32). In this application we would like to change the study design to between-group design, and to add two pain sensitivity tests.

We have now conducted a pilot study with 8 participants using endotoxin stimulation, but without fMRI, in a double-blind cross-over design in accordance with our prior ethical approval. In this testing session it became apparent that we are unable to blind the study for within-person comparisons using endotoxin vs placebo. The effect of the dose was exactly as expected and corresponded to the symptoms of early influenza, resolving in about 3 hours. Participants did not develop fever and heart rate and blood pressure were not affected. Since clear influenza symptoms occurred in about half of the participants we were unable to control for the anticipated effects when the subjects received endotoxin, and clearly reacted to it, on the first occasion. After the second session, it was clear to all subjects during which session they received endotoxin, since we had had a lengthy conversation concerning how they experienced the study, whether they felt secure, whether the information was sufficient and whether our “disease model” was too demanding or unpleasant.

To conduct the study as fairly as possible we would therefore like to make a few changes to the study protocol.

1. We believe that blinding participants to the study categories is incomplete and that the cognitive consequences of this are difficult to manage and may interfere with the study. The pain test is quite likely to be affected by anticipation of effects during both sessions. We would like to keep the same dose, since it yields the desired effect, but would like to switch from our current within-group design to a between-group design instead. **We are therefore applying for ethical approval to recruit twice as many subjects, 56 individuals in all, but to have each person participate on only one occasion.** **Remuneration will be SEK 1500.** Half of the subjects will receive endotoxin, the other half normal saline. We will inform everyone that they are to receive endotoxin in the same manner as before, and treat each test occasion as though we have injected endotoxin. As previously, only the responsible doctor will know what substance has been injected, and as before, the research supervisor will have minimal contact with the doctor. Everyone else working in the presence of the subject will be blinded as to category.

One primary aim of the study is to investigate whether the acute inflammatory response affects pain regulation. As expected, the various pain tests included in the pilot study differed somewhat, but overall, our findings suggest that endotoxin does have an effect. As previously stated, only those pain tests that demonstrate a definite effect from the endotoxin in the majority of pilot study participants will be used in the main study.

1. By definition, pain is a subjective experience. The response of our subjects includes a visual analogue scale (VAS) and we always explicitly ask individuals about their subjective experience here and now. In this context, however, more objective measures would also be desirable. One well validated measure is the lower limb flexion reflex (LLFR), also known as the nociceptive withdrawal reflex. This is a neurophysiological measure that provides information on spinal (i.e., spinal cord) and supraspinal (i.e., brain stem and brain) mechanisms concerning pain regulation, i.e. processes that are of interest in the current study. According to European Federation of Neurological Societies (EFNS) guidelines, the LLFR is the most commonly used of all nociceptive reflexes and appears to be the most reliable to assess treatment of pain [1].

*How does the Lower Limb Flexion Reflex test work?*

Two surface electrodes designed for the purpose are applied to the washed skin above the sural nerve on the outside of one foot. These are connected using a safety switch to the Biopac STMISOL device, designed and approved for human experiments for this purpose and similar applications. The system has an array of safety features and is of course completely isolated from external power sources. Each stimulus consists of a sequence of rectangular pulses at a frequency of 100–300 Hz over a few milliseconds. Surface sensors (2 + one ground) for electromyographic (EMG) measurement of the reflex are attached with adhesive to the lower aspect of the thigh muscle (biceps femoris muscle). A simple titration, beginning with a current of 0 mA and increasing by between about 2 and 4 mA per step, is carried out according to a commonly used step-wise algorithm to ascertain the relevant subject’s threshold for eliciting the reflex. Following this, a number of suprathreshold stimuli are applied according to standard methodology and muscle response is measured using EMG.

One measurement prior to and after each endotoxin/control injection is planned. We have acquired substantial experience of this method in Prof. Martin Ingvar’s group, where promising results using this technique in a recently concluded study are currently being compiled. The total time required for each pain test will be about 30 minutes.

1. Data from animal experiments suggest that TNF-α maintained inflammation contributes to the occurrence of neuropathic pain [2]. One model that has been proposed, which to some extent is similar to the burning sensation that is a component of such pain, is Thunberg’s illusion (also known as Thermal Grill Illusion). The illusion occurs when warm (about 42 ^o^C) and cool (about 16 ^o^C) elements, respectively, that are not inherently painful are placed so they overlap, which is experienced as mild burning discomfort. Thunberg’s illusion has been suggested as a suitable model for certain aspects of neuropathic pain [3]. The burning sensation is tolerable, safe and instantaneously reversible when the hand/forearm is removed from the thermode. Degree of discomfort is rated using a scale such as VAS. Simple pain assessment using this illusion in the relevant inflammation model can increase understanding about the processes that occur on a spinal and cortical level in the interaction between pain and inflammation.  Within our group we have acquired expertise on this type of testing and have a suitable thermode made by a Swedish medical device company that is appropriate for the purpose.  This test will be carried out after the conclusion of the main study. The test will take place during the time when the subject remains in the hospital for post-experimental monitoring.

In summary, we are applying for ethical approval for the following changes to the current study:

1. Between-group design to replace within-group design to make blinding possible and to avoid anticipating effects that may affect the results.
2. Addition of lower limb flexion test for the purpose of measuring pain responses more objectively.
3. Addition of thermal grill illusion to investigate whether acute immune activation has any effect on systems relevant to neuropathic pain.

References:

1. Sandrini, G., et al., The lower limb flexion reflex in humans. Prog Neurobiol, 2005. 77(6): p. 353–95.

2. Leung, L. and C.M. Cahill, TNF-alpha and neuropathic pain--a review. J Neuroinflammation. 7: p. 27.

3. Craig, A.D., Editorial: Can the basis for central neuropathic pain be identified

by using a thermal grill? Pain, 2008. 135: p. 215–216.


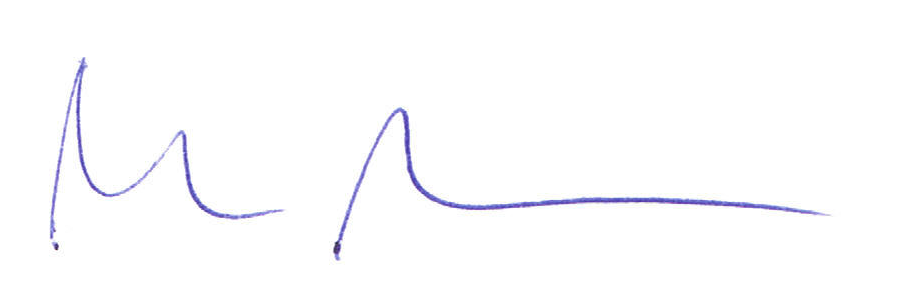
 Stockholm, 23 September 2010

----------------------------------------------- -----------------------------------------

Mats Lekander, associate professor, project coordinator Place, date
